# Versioning biological cells for trustworthy cell engineering

**DOI:** 10.1038/s41467-022-28350-4

**Published:** 2022-02-09

**Authors:** Jonathan Tellechea-Luzardo, Leanne Hobbs, Elena Velázquez, Lenka Pelechova, Simon Woods, Víctor de Lorenzo, Natalio Krasnogor

**Affiliations:** 1 Interdisciplinary Computing and Complex Biosystems (ICOS) Research Group, Newcastle University, Newcastle Upon Tyne, NE4 5TG UK; 2 Systems and Synthetic Biology Department, Centro Nacional de Biotecnología (CNB-CSIC), 28049 Madrid, Spain; 3 Policy Ethics and Life Sciences (PEALS), Newcastle University, Newcastle Upon Tyne, NE1 7RU UK

**Keywords:** Biotechnology, Communication and replication, Synthetic biology

## Abstract

“Full-stack” biotechnology platforms for cell line (re)programming are on the horizon, thanks mostly to (a) advances in gene synthesis and editing techniques as well as (b) the growing integration of life science research with informatics, the internet of things and automation. These emerging platforms will accelerate the production and consumption of biological products. Hence, traceability, transparency, and—ultimately—trustworthiness is required from cradle to grave for engineered cell lines and their engineering processes. Here we report a cloud-based version control system for biotechnology that (a) keeps track and organizes the digital data produced during cell engineering and (b) molecularly links that data to the associated living samples. Barcoding protocols, based on standard genetic engineering methods, to molecularly link to the cloud-based version control system six species, including gram-negative and gram-positive bacteria as well as eukaryote cells, are shown. We argue that version control for cell engineering marks a significant step toward more open, reproducible, easier to trace and share, and more trustworthy engineering biology.

## Abstract (web summary)

Full traceability and transparency are important to establish trust in engineered cell lines. Here the authors argue that version control for cell engineering marks a significant step toward more open, reproducible, traceable and ultimately more trustworthy engineering biology.

## Introduction

Engineering biology is exploding with advances ranging from new genome editing tools^1^, to genetically encodable materials for advanced sensing of cells physiological states, electrical fields and mechanical stresses^2,3^, programmable and functional microbial-based living materials^4^, environmental remediation and pollution control^5^ to advanced in vivo data storage^6^. Moreover, these advances in fundamental science are rapidly translating into new companies^7^ and consumer products^8^, which within the first half of this century, are set to impact most areas of our lives.

Perhaps the most convincing example of the pace of progress is the global scientific response to the current SARS-CoV-2 pandemic. In a matter of weeks after detecting the outbreak, the virus was isolated, its genome sequenced and published^9^ and made available for research. Slightly less than a year later multiple vaccines were already being deployed to combat the virus. This would have been unimaginable just 10 years ago. Although this is an extreme example arising out of an extreme situation, it is to be expected that with the commoditization of synthetic DNA and the wider availability of powerful gene-editing tools^10^, the number of engineered strains will rapidly increase. Indeed, cheaper DNA synthesis technology and the development of high throughput, automated, cloning processes allows the creation of large plasmid and combinatorial DNA libraries^11,12^ in a matter of days, including the modification of recalcitrant species’ genomes^13^, which previously were difficult to edit.

And yet, while engineering biology has changed profoundly in the last few years, there are still deep gaps in the way the process of strain engineering is done and disseminated. For example, engineering a “synthetic biology agent”^14^ produces large quantities of information: published articles, protocols, notebooks, models, databases, sequencing and other types of data (e.g., metabolomics, proteomics, lipidomics, etc.). Combined, all these information sources may add up to terabytes of data but only a relatively small percentage of it is being made available when results are published in specialized outlets. This gap in scientific practice has led to an ongoing crisis in cell line misidentification^15–17^, a recognized lack of reproducibility^18^, sometimes causing high profile retractions^19^ and often resulting in weakening public attitudes to new and emerging technologies.

It is thus clear that this gap in scientific practice requires a response on multiple fronts, to which this paper contributes in a number of practical ways with the introduction of CellRepo as a community resource.

**Fig. 1:**
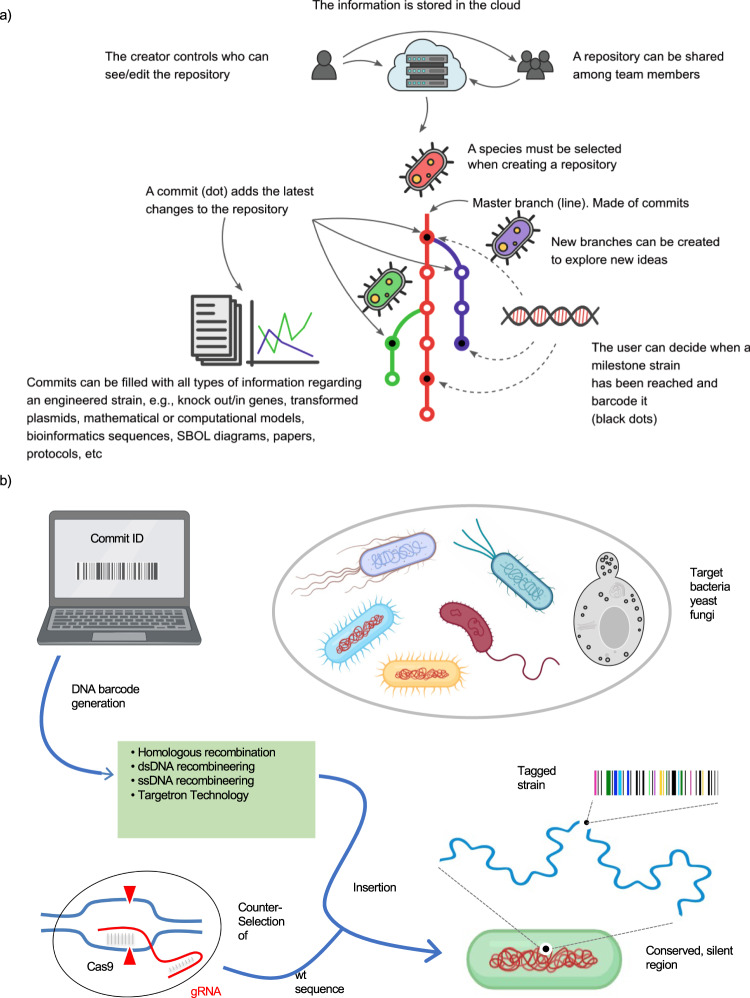
Version control and barcoding of engineered cells. **a** Graphical description of CellRepo. A repository contains all the information of the project. Several ideas can be tested using the branching mechanism. The users can decide what to document at each stage in the project through commits. At user-defined steps, a physical DNA barcode can be generated to be inserted in the genome of the strain. **b** Workflow for barcoding any microorganism or strain of interest. The roadmap can be applied to basically any biological system amenable to genomic insertions of short DNA sequences. Once a specific barcode is committed, it is synthesized and delivered to a stable region of the genome of the target organism. The different approaches followed in this study to select appropriate barcoding locations are described in the Supplementary Material. This can be made with a whole collection of genetic tools available to this end in a fashion dependent or independent on homologous recombination. As the resulting insertion is expected not to generate a conspicuous phenotype, proper delivery of the barcode to the expected genomic site is secured and selected with CRISPR/Cas9 technology or via the more traditional antibiotic resistance or auxotrophic marker approach depending on the laboratory carrying out the barcoding.

CellRepo is a version control system for cell engineering. Version control is the practice of monitoring modifications in the source code of computer programs or other (digital) objects, which is assisted by the use of special software that keeps track of the code, its changes over time, the changes’ authors and other metadata. CellRepo integrates, on the one hand, a cloud-based version control software for tracking cell lines’ digital footprints and, on the other hand, living samples’ molecular barcoding protocols to link the biological sample back to their cloud digital information. CellRepo relies on two fundamental pillars. Firstly, during the process of engineering a new strain, changes introduced to the cell line are recorded in “commits” that include information such as the genotype, phenotype, author of the modifications, laboratory protocols, characterization profiles, etc. (Fig. 1a). The history of commits tracks the digital footprint produced during the process of cell engineering. The second pillar is the physical linking of a living sample to a commit via the chromosomal introduction of a unique barcode related to the commit (Fig. 1b).

Genomic barcodes have been recently recommended as the way forward for tagging^14,20^ synthetic biology chassis with unique identifiers for the sake of traceability, intellectual property issues and environmental risk assessment (ERA)^21^. Current schemes for curbing the propagation of genetically modified microorganisms with genetic firewalls or conditional killing systems are still insufficient to guarantee certainty of containment^22^. In this context, barcodes appear as either an alternative or as a complement to such firewalls for a sound ERA of agents designed for deliberate release—or accidentally escaped thereof. Genomically inserted identifiers instantly refer to digital twins with all available documentation on the live construct at stake (see below), not only in terms of species and genetic pedigree but also regarding safety aspects and indications for countermeasures in case of undesirable propagation. In that sense, barcodes may ease the current emphasis on containment toward a more realistic scenario of management^23^, thereby facilitating the regulatory and approval process^14,21^.

To demonstrate the wide applicability of CellRepo, we show how the platform—in conjunction with well-established peer-reviewed protocols to genetically engineer various organisms—can be applied to six of the most important and diverse microbial species used in both academia and industry (*Escherichia coli*, *Bacillus subtilis*, *Streptomyces albidoflavus*, *Pseudomonas putida*, *Saccharomyces cerevisiae* and *Komagataella phaffii*—previously known as *Pichia pastoris*). It is expected that the same principles can be applied to more, if not all, species which have been already domesticated and engineered.

## Results

### CellRepo is a cloud version control for engineering biology

**Fig. 2:**
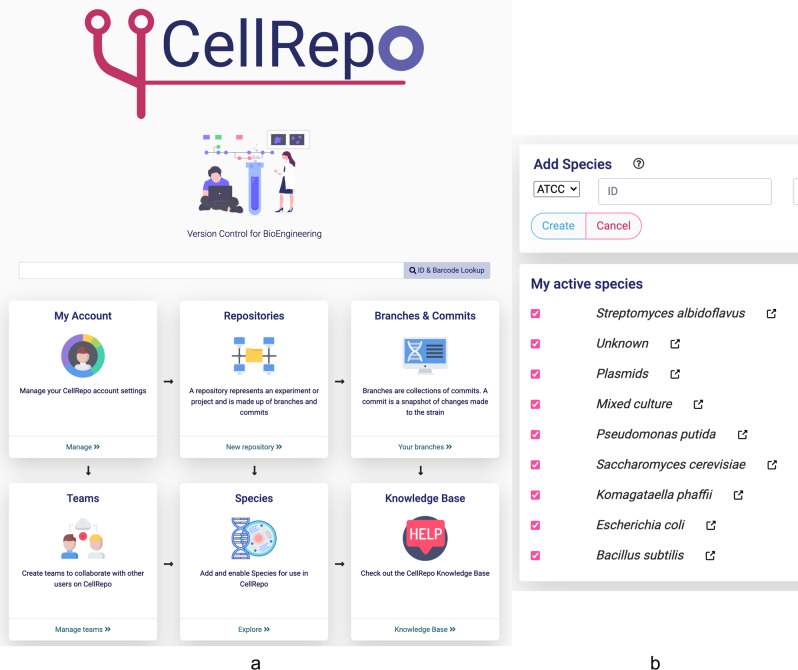
CellRepo workspace. **a** Homepage after a user signs in. From there they can search and browse their own strain repositories or those they participate as a team member. Also, they have access to any strain repository that has been made public. Users can also create new version control repositories, make commits to them, add new species, etc. The landing page also shows a recent activity registry of the users and the repositories they have access to. **b** Species search functionality: users can look up a species database and select the ones they want to use as a base for a cell engineering project. If a species is not available in the database users can make a request to add it.

We created a cloud-based community resource built on top of a modern software engineering stack for web applications. As in any cloud-based application, the user needs to register, providing a name, e-mail address and password. An avatar picture can be uploaded to personalize the experience and to be more recognizable by other users (e.g., collaborators). After registering, the user will receive a confirmation by e-mail. Finally, the user can sign in by typing the registration e-mail and password. Once a user signs in, they land on the homepage (Fig. 2a) that contains everything needed to build repositories of engineered strains, manage their accounts and the teams they work with. From the initial page, it is also possible to access the system documentation (“Knowledge Base”). The blue upper horizontal quick menu links to all the aforementioned features and is present on every page on the website. This menu also contains a search bar. This allows users to look for repositories and commits accessible to them (i.e., cell repositories they own, that belong to their teams or public cell repositories) and look for identifiers to find the documentation on specific strains/plasmids.

The first step to start a repository is to select a species. The server is linked to up-to-date databases of organisms (Fig. 2b). This ensures that the users are always able to use the species they need and that these are well documented. To ease the finding of new species to work with, the users need to pre-select them from the database and add the species to their unique list of in-use organisms.

Repositories are projects or experiments (e.g., compound production, protein expression, etc.) and are usually linked to a specific species. Metadata information like the name and description of the project, as well as information about the purpose or how to use a specific strain repository, can be added. Repositories may have different “visibilities”: public (anyone can see the content of the repository), team (visible just for members of the same team or laboratory) and private (just the user can see and add changes). A user may change its repository visibility at any point in time. A repository can have many branches and in turn, branches are made of commits. The name of the initial branch can be set during the creation of the repository.

**Fig. 3:**
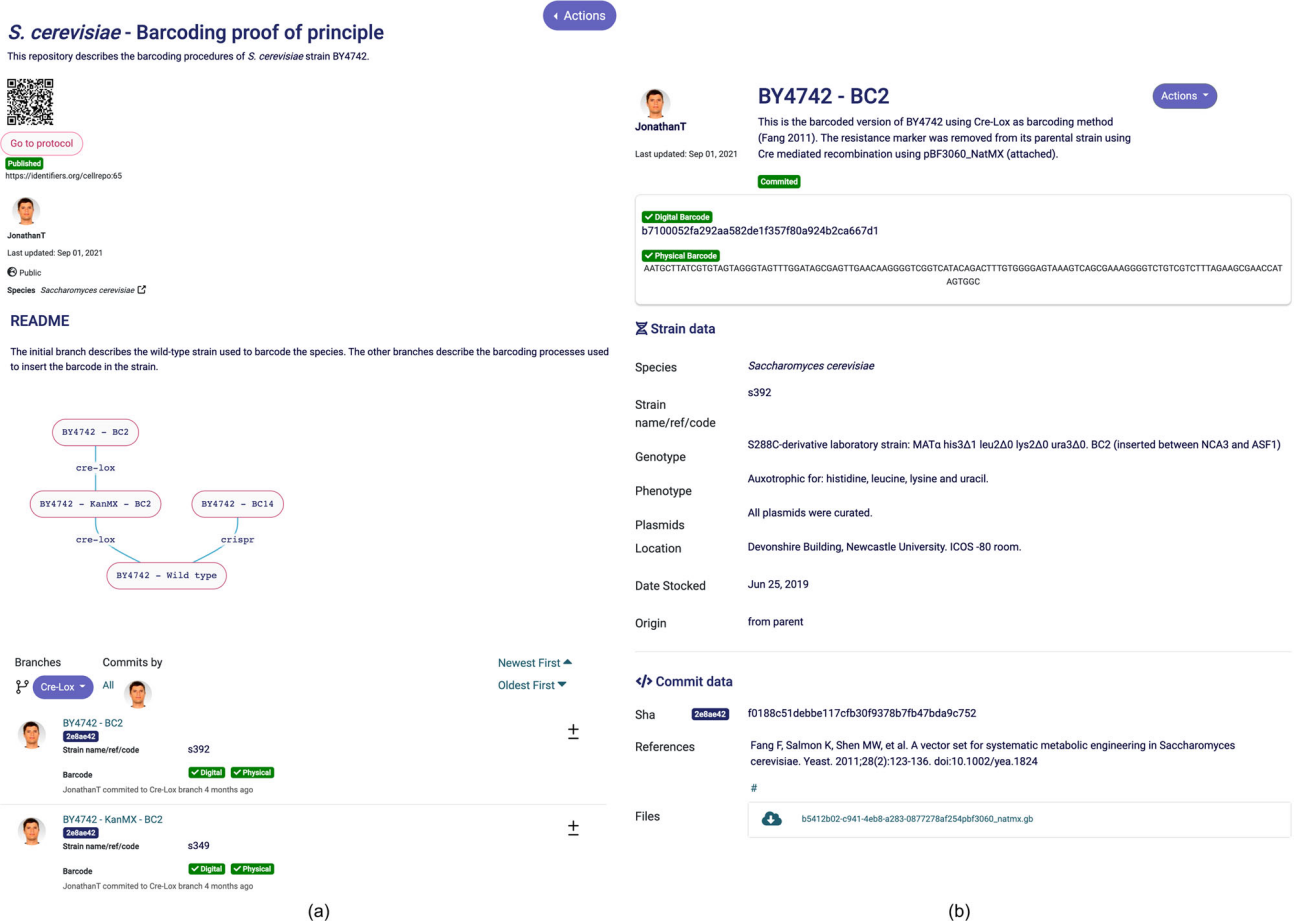
Cell repository details. **a** Strain Engineering Repositories contain all the digital footprint produced during the engineering of a cell line. The repository provides general information about the cell line project (in this example, the barcoding proof of principle of *S. cerevisiae*). It also contains all the different “commits” that were made during the engineering process. **b** A commit represents a related set of changes introduced into a cell line. All commits have a unique digital identifier and some commits (decided by the user) may also have a physical identifier barcode that is physically inserted into the cell chromosome. Recovering the barcode by sequencing allows a cell engineer to recover the id of the commit containing all the digital footprint of the cell line.

A repository (Fig. 3a) has a main or leader branch (named during the repository creation) and many other branches. Each branch represents a new direction or idea the users want to pursue in their cell engineering activities (e.g., novel protocols, different gene edition order, etc.).

A commit (Fig. 3b) captures the status of the engineered strain at a specific point in time. The amount of information contained in a commit is up to the user (it can be as simple as a new strain name or as complex as a brand-new strain creation by modifying the genome and adding several documents). In addition, the commits are the containers of the uploaded documentation (which can be in the form of documents, models, sequences, etc.).

Once in a repository, the user can choose a branch to commit. The “new commit” button opens a form in which various types of information can be inserted. For example, the user can name and describe the commit (what is being done? why? what for?). Importantly, all types of documentation (supporting the commit) can be uploaded on this page such as construct sequence, electrophoresis gel pictures, SBOL^24^ files, growths and fluorescence curves, sequencing results, automation worklist instructions, computer models, etc. The user can also provide genotype and phenotype information, the storage location of the strain, safety information, acceptable material transfer agreements for the strain, etc.

The user can choose the level of granularity of commits that best fits its laboratory practice, e.g., a commit might represent a single cell modification or multiple multi-loci genetic changes.

When creating a new commit, the user can decide whether the change is important enough (e.g., a milestone) to be physically barcoded into the cell. If that is the case, the system allows the generation of a unique barcode sequence. The barcode can then be synthesized and inserted into the strain. Once created, the commit will be linked unequivocally to the strain carrying the barcode sequence.

CellRepo allows users to be part of collaboration “teams” for cell engineering. Team members of a strain repository can make commits and create new branches to the cell line history. Furthermore, teams of researchers can share repositories, track strains and be up to date on the experiments being carried out in their projects. Creating a new team is as easy as providing a name to the team and adding CellRepo users to it. Once established, it is possible to see all the members and the shared repositories and keep track of the activity taking place on the repository.

### In vivo barcoding experiments

Different barcoding protocols (detailed step-by-step protocols can be found in the extensive Supplementary material) were assessed for *E. coli*, *B. subtilis*, *S. albidoflavus*, *P. putida*, *S. cerevisiae* and *K. phaffii*—previously known as *P. pastoris*. These protocols are used to introduce into the chromosome of the cell the barcodes that are automatically generated by CellRepo when a user creates a new commit in the version control system. CellRepo maps the unique commit identifier into DNA sequences that are then used as barcodes. All the tested barcoding procedures successfully barcoded the target species (Supplementary Table 5). URL links and QR codes for all CellRepo repositories for these experiments can be found in Supplementary Table 6.

### For all species tested, barcodes are genetically, physiologically innocuous and stable over a range of growth conditions

Barcoding a strain should have little to no effect on its growth profile; growth profiles of barcoded cells were compared to wild-type (i.e., non-barcoded) strains (Supplementary Fig. 5).

The six growth profiles show no significant differences between barcoded strains and the corresponding non-barcoded parental strains. This confirms that the barcode insertion has little effect on the growth of the different species.

We also evaluated whether or not the barcoding protocols introduced unplanned mutations in the recipient cell. For instance, this can help choose a specific barcoding method over another. To do this, the whole genome of the barcoded strains and the wild-type strains used was sequenced. The results can be found in Supplementary Tables 7–12.

*E. coli* results show that the three clones barcoded using Lambda-Red method had the same point mutation in an intergenic region (Supplementary Table 7). This may be explained by the fact that the initial colony chosen to start the insertion process already contained the mutation or it was acquired during the process. In any case, the mutation is intergenic and does not seem to affect the cells. In one of the clones barcoded using gRNA1, two-point mutations appear in different CDS. Strains barcoded using gRNA2 do not show any mutation.

*B. subtilis* was barcoded using three different methods. Both CRISPR (only one gRNA tested) and Toxin-mediated barcoded cells show no mutations in all the different clones. In one of the barcoded strains using Cre-Lox, a mutation appears. In a different clone, two different SNPs could be detected. All the mutations are in different CDS (Supplementary Table 8).

For *P. putida*, two clones were barcoded using the CRISPR/targetron system and both showed one mutation in different CDS (Supplementary Table 9).

*S. cerevisiae* was barcoded using two methods. In both of them, most of the mutations that appear are tandem repeat related. These could be acquired during the insertion process or could be sequencing artifacts related to this type of repetitive sequence. Two strains barcoded using Cre-Lox show single point mutations. Four mutations (tandem repeats) are observed in Strain 2 (CRISPR). A single point SNP can be observed in Strain 3 (Supplementary Table 10).

One of the two strains of *K. phaffii* shows two-point mutations in intergenic regions (Supplementary Table 11).

Finally, *S. albidoflavus* was barcoded using CRISPR. NGS analysis of *S. albidoflavus* shows a larger number of variants. Strain 1 barcoded with gRNA1 shows two different base-pair changes in different CDS. However, no mutations were found for Strain 2 (gRNA1) (Supplementary Table 12). The three strains produced using gRNA2 count six, three and two SNPs, respectively. In eukaryotes, CRISPR-caused double-strand breaks (DSB) can be repaired by non-homologous end joining (NHEJ) or homologous recombination (HR) in the presence of a repair template. NHEJ repair is usually imprecise and indels occur. In the case of *S. cerevisiae*, however, it has been observed that NHEJ hugely decreases cell survival and, when a repair template is provided, HR is the prevalent repair mechanism in this species^25^. Prokaryotes, on the other hand, usually lack NHEJ repair mechanisms. Nevertheless, it has been described in *Streptomyces coelicolor* among other bacterial species. In this Actinobacteria, closely related to *S. albidoflavus*, researchers knocked out genes using CRISPR without providing a repair template and allowing the native NHEJ system to—wrongly—repair the DSB^26^. It may be possible that the gRNAs caused CRISPR off-target activity that was repaired by NHEJ causing mutations to appear. Together with the fact that the genome of *S. albidoflavus* has a high GC content and produced the worst sequencing quality of the analyzed species, this may explain the higher mutation count. Even though other mechanisms may explain the observed variation (see next), users wanting to use pSA-CRISPR-gRNA2 should bear in mind these extra mutations.

The NGS analysis shows that for all the sequenced strains the number of total mutations found in each method is low. The mutations are not constant in the different replicas sent to sequencing. We hypothesize that the mutations (if not sequencing artifacts) are caused by the natural mutation rate in each species during several cycles of growth (both in liquid and solid media). This is supported by^26^. In this *S. coelicolor* CRISPR edition experiment, the control strain, in which an empty (no target) gRNA was provided to the cells caused a total of seven mutations, three of which were in coding regions. Similar results were found in a CRISPR experiment in *S. cerevisiae* where they detected 10 SNPs that were probably caused by the successive transformation rounds required for the experiment^27^.

Importantly, we found no structural variants in any of the sequenced strains.

The NGS analysis suggests that the barcoding procedures do not change the genome of the strains more than what would be expected while carrying out conventional genetic engineering protocols. CellRepo users can use this information to choose the barcoding method specific for each species that best fits them.

We also carried out stability evaluation of the barcodes where the stability of the barcode sequences was assessed under five different growth conditions. The barcodes were stable both in terms of presence (Supplementary Table 13) and sequence integrity after the long-term experiments (Supplementary Figs. 6–35). Finally, the usage of barcoded strains as a way to track the dissemination of GMOs is described (Supplementary Fig. 36). In the particular case of a gene drive (which has been proposed as a solution to some infectious diseases transmitted to humans from animal and insect vectors), barcode sequences could pinpoint the source of the released modified organisms (29) (intentionally or accidentally) in the environment.

### Barcode survival after long-term growth

Stationary phase mutagenesis occurs to microorganisms when they are deprived of nutrients. Mutations may arise without active cell division or global DNA replication^28,29^. This phenomenon has been demonstrated in *E. coli*^30,31^, *B. subtilis*^32^, *P. putida*^33^ and *S. cerevisiae*^34,35^. Because of that, we evaluated whether the barcoding DNA sequence introduced is stable during continuous stationary phase growth and other non-exponential growth profiles, common in laboratory and industrial processes like batch fermentation growth and restreaks on solid media.

To assess that the barcode stays in the insertion site and that its sequence is still retrievable even after long periods of growth, we ran on all six species five different experiments for 10 days.

As a preliminary experiment, for each condition, the final day single colonies were restreaked and the barcode region was PCR amplified and sent to Sanger sequencing (Eurofins Genomics). For all the colonies tested, we were able to confirm the barcode presence by PCR in all the cases. Supplementary Table 13 describes in detail the sequencing results of this experiment.

To have a more thorough view of what happened to the barcode sequence during the long-term experiments, an NGS analysis of the PCR purified product of the barcoded region of the cell population in conditions 1–4 was performed.

Mutation analysis of the barcode sequences for all species can be found in Supplementary Figs. 6–35.

*E. coli* control (Supplementary Fig. 6) showed a base-pair change in 50% of the reads. To check if the glycerol stock had any mutation, ten colonies were isolated and the PCR product of the barcode region was sent to Sanger sequencing. No mutations were found. A point mutation in the initial PCR cycles of the reaction sent to NGS explains this result.

The percentage of reads showing either INDELs or base-pair changes stayed at the same value as the one observed in the control experiment (lower than 0.05%).

Both the single colonies and the population level experiments show that the barcode was still present after the long-term incubation period and that the sequence was stable on all five experimental conditions.

### Barcodes provide a backtrack signal for GMO dispersion experiments

Gene drive technology allows the researchers to propagate a specific genetic modification through a population^36,37^. The scientific community needs to assess the risk of this kind of research. Barcode sequences can be helpful in this matter and uniquely identify the laboratories where a gene drive experiment was carried out, the purpose of the modification and any other relevant data (e.g., safety measures implemented).

Supplementary Fig. S36a graphically describes the gene drive molecular mechanism. The barcode identifier was coupled with the intended modification (*ADE2* deletion cassette). Supplementary Fig. 36b shows that the barcoded cells (red pigment) can survive in SC-Uracil media. Haploid cells coming from the unmodified parent cell show red pigment only when pCas9 plasmid was also present. In all cases, it was possible to PCR and sequence the barcode sequence from each haploid individual.

## Discussion

Version control has been a pillar of software engineering and—notwithstanding that strain engineering is a very different discipline than software engineering—we believe that wider adoption of version control principles could substantially improve the quality of research that relies on modifying and engineering cell lines. We thus postulate that adoption of CellRepo will improve:

- Traceability: by physically linking a cell line chromosome to a commit id in the cloud, one can know the exact documentation for a strain. Besides technical information about the cell line, stored information also includes the intention behind genetic changes and allows proper allocation of credit (who created a particular commit in a cell line) for work done in the laboratory.
- Responsibility: because key cell lines can be tracked, branched, audited and ownership assigned both digitally and molecularly provenance, quality assurance and trustworthiness are enhanced.
- Reproducibility: it will be easier to reproduce experiments and avoid false leads because one will have a complete long-term change history of every modification to cell lines of interest. This change history includes the author of the change, laboratory of origin, the date of the change and written notes on the purpose and intention of each modification. Having a complete history of cell line modification also provides the ability to “revert” back to previous versions of a cell line, which is great for bug fixing in software engineering, and we believe will be useful in cell engineering too. As biologists, this would mean knowing exactly what someone else did at each commit-able stage in a project. Furthermore, this enables to base, with ease and confidence, a new cell line project on trustworthy pre-existing repositories and thus absorbing the history that came before it.
- Collaboration: CellRepo improves collaboration. Version control systems allow complex software to be written by single individuals as well as by remote teams. Similarly. CellRepo accommodates both single or multi scientist projects, maintaining a clear record of contributions. Furthermore, CellRepo does not force new workflows into laboratory users. Rather it is agnostic to the specific tools they already use and can accommodate uploads from any laboratory tools they may already be using. CellRepo also allows fine-tuning of a cell engineering project visibility by allowing repositories to be entirely private, shareable or public.
- Transparency: our proposed version control system for cell engineering calls for more transparency in the process of making science. As we argued, research ought to be transparent and transparency benefits internal teamwork and enhances public trust in science. CellRepo empowers the sharing of cell line repositories in the same way that version control systems such as GitHub, Bitbucket or Gitlab host and promote open source projects. Like with open source projects, each snapshot of a cell repository shows the “good, the bad, and the ugly” of each stage in the development of a cell line. Through transparency, “bugs in the bugs” would be more readily discovered and corrected. Furthermore, as recently argued^38^, there are two growing trends in science. One seeks to make science more open and the other more reproducible, but the adherents of these two trends do not always work concurrently toward openness and reproducibility. We believe our paper is a step toward bridging these two camps.
- Economics: in software engineering, it is possible to develop software without using any version control. However, doing so subjects project owners to data and source code loss risk, loss of project history and the inability to collaborate in real-time. No professional software engineering team should or would accept those risks. Thus, we expect that as CellRepo become the norm in life sciences, important economic benefits will become more tangible.

We have adapted recombineering protocols to barcoding for version control in four bacterial species and two fungal species thus, in principle, one can create a truly universal tracking system for all lab-made cell lines. The barcodes introduced into key cell engineering milestones are the commit ids from the version control system bio-orthogonally mapped to DNA. We note that these barcodes fulfill a different role than whole-genome sequencing of a milestone, and hence cannot be solely replaced by it. For example, two different cell engineering projects might start from the same strain (hence having the same genome sequence) but require that they be distinguished from each other: different teams and laboratories, different goals and objectives for the project, different material transfer agreements or IP regimes, etc. Barcodes, watermarks and similar digital signatures embedded in the genome can be used to implement more sophisticated “digital rights management” that genome data by itself cannot. Moreover, although whole-genome sequencing is becoming cheaper, it is still a far more expensive and complex process than sequencing a relatively short barcode as we propose here. Altogether, our work demonstrates that barcoding technology can be applied to many industrially and academically relevant microbial species; the barcode sequences are stable under laboratory conditions, they do not affect the growth of the barcoded strains and they can be used as a backtrack signal during GMO dispersion experiments. In the future, other kingdoms of life will also be added to CellRepo and tested in similar ways. Indeed, mammalian cells can be added straight away to CellRepo without a barcode or they could be barcoded via CRISPR/CAS methods or using lentiviral routes.

Importantly, we believe that a more traceable, reproducible and transparent development of engineered cell lines will contribute to improved public attitudes to the discipline. Indeed, public attitudes toward science in general, and in particular newly emerging technologies such as engineering biology, have been the focus of study over the last few decades. Some of the earlier studies were based on the so-called deficit model that assumes that the publics’ support of science and novel technologies is built on their knowledge of science. Although this model is still used by some scientists, surveys and polls have shown that more knowledge of science (or technology) does not necessarily lead to more public support^39,40^. It was also shown that deference to science^41^ had a large impact on perceptions of, e.g., nanotechnology and that public trust in the claims made by experts or by scientific institutions^42,43^ is an important factor in understanding the publics’ attitudes to science. Similarly, although general support was found for the then-emerging field of synthetic biology^44^, it was tempered by fears over misuse, health and environmental impacts, control and governance. Taken together these findings suggest that the publics’ concerns about engineering biology rest more with the method and processes underpinning the research rather than in the better understanding of one or another specific technical advance. That is, transparency and accountability of the research are the main concern in the publics’ attitudes to engineering biology^44–46^.

Moreover, the potential for public mistrust in innovative science has been echoed in the science community’s crisis of conscience about the integrity of science itself^47–49^. Similar concerns have been raised by other studies, UK Research and Innovation/Research England’s survey on research integrity^50^ confirmed that open and transparent research is regarded as central to rigor, reproducibility, and public trust. Commentators on the “crisis” broadly agree that the research community is overwhelmingly motivated by these values but that other factors within the research culture can make it difficult to uphold these principles (e.g., see^51^). In response, there have been moves to promote measures that enable transparency, reproducibility and openness. For example, the “FAIR Principles”^52^, namely findable, accessible, interoperable and re-usable, have been widely adopted while the UKRI Research Concordat on Open Research Data^53^ has been adopted by multiple stakeholders within the UK research community, with similar schemes elsewhere. The issues just highlighted also affect engineering biology and life sciences more generally.

Engineering biology, and in particular, strain engineering is hard, but the community working on cell engineering makes it harder by not adequately documenting, tracking and sharing the process of genetic engineering. Baker describes^54^ the important contribution quality assurance processes can make to research integrity by enabling reproducibility and avoiding the opportunity for cherry-picking of results and data massaging. With this spirit in mind, in this paper, we have introduced a version control system for cell engineering. Versioning biological cells will lead to more trustworthy cell engineering.

## Methods

### Repository access

CellRepo can be accessed from https://cellrepo.ico2s.org

### Creating or searching for a CellRepo repository

Repositories stored in CellRepo contain the entire digital footprint of a strain engineering process, which is linked to the cells in question via their genome stored barcode. Each repository represents an experiment/project on a cell line. To create a new repository, the user must navigate to the “Repositories” page using the navigation bar at the top of the page or the card on the homepage. Click the “Add” button to create a new repository. The repository can now be named and described in the form and the visibility of the project can be selected. The species of the repository can be chosen from a list of pre-selected species; if the desired species is not available in any of the linked databases then the user can request the addition of the new species. Finally, the repository can be created by clicking the “Create” button.

The user can also choose to start their experiments from a “branch” of another repository. To do this, they can search for project keywords or, importantly, barcode sequences using the “Search” functionality and create a new branch or commit from that point onwards.

**Fig. 4:**
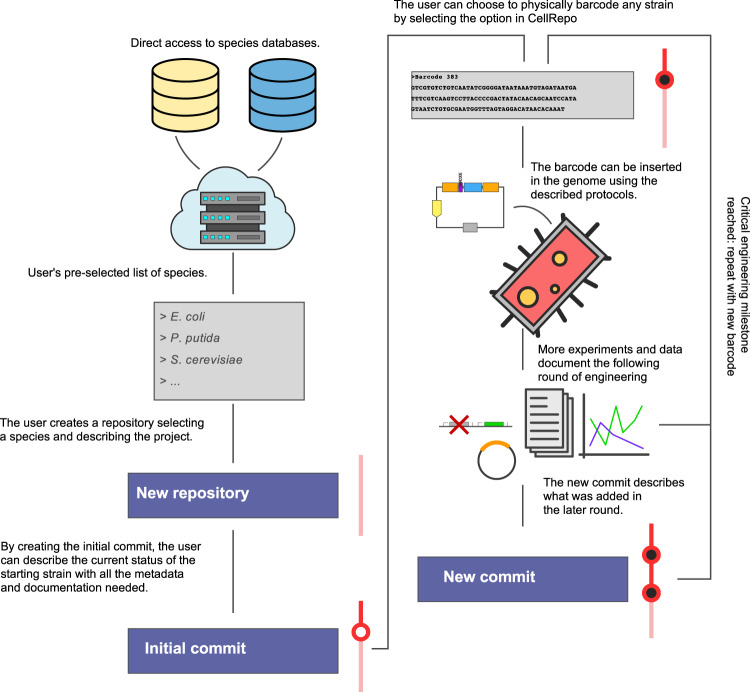
CellRepo usage workflow. The user can choose from species directly linked from public databases. A new repository must be named and described to host the documentation of a project. Once created, the repository can be filled with commits to document the history of the strain. When a milestone strain is reached, the user can choose to generate a DNA barcode to be inserted in the genome of the strain.

Once the repository is created, the user can now start building their project using commits (see Fig. 4). Each commit adds the latest changes to the repository. It contains information about the changes to the cell line, who did it, references, barcodes, documents etc. Commits are a representation of a cell line at an exact moment in time. To create new commits, select “New Commit” from the Actions menu to add a commit to the repository. Fill in the commit form with information about your change. Add any files you want to upload that supplement the commit. At this step, the user can choose to add a DNA barcode by ticking a box. After synthesis, the DNA barcode can now be inserted into the strain’s genome by the protocols described in the Supplementary Information.

After more experiments are carried out on the strain, the repo can be updated, more information and documentation added, and the barcode sequence can be updated in the genome. These steps can be repeated as many times as necessary to document the history of the strain and the project.

#### Barcoding site selection

*E. coli* and *B. subtilis* have known lists of essential genes^55,56^. Using these lists, it was possible to create a simple python script to get possible candidates of essential gene pairs. The script used as input the GFF3 annotation file of the strain and the list of the essential genes. Both files had to be curated to obtain a uniform gene name nomenclature. As output, the algorithm gives back a pair of essential genes next to each other, the orientation of both genes and the DNA sequence that separates them. Then, databases^57^ and prediction tools^58,59^ were used to check for the presence of regulatory elements in the intergenic sequences that did not appear in the annotation files. Once a good candidate pair was obtained, the target region was aligned against the most common laboratory strains of the specific species to check for the presence of the possible barcoding region in them.

No list of core essential genes for *P. putida* strains was found in the literature except for conditional essential genes in some conditions^60^ and essential genes in the related species *Pseudomonas aeruginosa*^61–63^. For this reason, well-known generally conserved essential genes were taken into consideration to choose one possible insertion locus for the barcode. *glmS* gene was chosen as a good candidate as it is a broad-host conserved gene in many species and it has a long enough intergenic region for the insertion of Ll.LtrB intron carrying barcodes. The procedure to select the exact insertion site inside the intergenic region downstream of *glmS* was adapted from^64^. In general, the PP5408-glmS intergenic region was surveyed for good Ll.LtrB intron insertion sites in the Clostron.com website. The sequences needed for tn7 insertion were avoided as we did not want to hinder the possibility of using this insertion method before or after the barcoding procedure. From the retrieved list of insertion loci, one was chosen from previous data verifying the correct insertion of Ll.LtrB in this site^65^.

Similarly, to *P. putida*, no essential gene information was found for *S. albidoflavus* (previously known as *Streptomyces albus*). However, in this case, we wanted to follow a different approach to showcase the flexibility of the proposed system for new candidate species to be barcoded. A close relative, *S. coelicolor*, is the model species for the study of the Streptomyces genus. For this species, there is a genome-scale metabolic model with gene essentiality data available that can be applied to other *Streptomycetes*^66^. Using this model, just reactions catalyzed by a known *S. coelicolor* gene, essential for all conditions tested and with no isoenzymes were considered. Using this data, it was possible to create a list of putative essential genes (Supplementary Table 1) for *S. albidoflavus* by aligning each of the *S. coelicolor* A3(2) essential genes to *S. albidoflavus* J1074 database. Feeding this list to the script described found no good candidate pair. The number of essential genes next to each other was too low and the few candidate pairs found had problematic intergenic regions. A simpler approach was followed.

Using the list of putative essential genes for J1074, the whole genome was analyzed looking for clusters of nearby essential genes. In this case, the resulting candidate pair genes were not next to each other but separated by one or more non-essential genes.

To check if the putative essential gene pair was conserved among different Streptomyces species, the protein sequences of the chosen candidate genes were aligned against the Streptomyces protein database using BLAST to check if the genes were conserved among different species (Supplementary Fig. 1). The results suggest that the two selected genes are conserved and are good candidates for essentiality.

*S. cerevisiae* is well known among the synthetic biology community and there is plenty of well-curated information about it. The user of CellRepo could choose to insert the barcode sequence into an already known and curated insertion site. These sites are used for example in microbial cell factories experiments to insert heterologous genes. By using this type of site, the possibility to barcode a strain, while the user’s desired edition occurs is shown feasible. It was decided to go for an already known insertion site flanked by essential genes used in microbial cell factories experiments^67^. Also, this site has previously been used to test CRISPR plasmids set in *S. cerevisiae*^68^.

*K. phaffii* (previously known as *P. pastoris*) is known for its ability to produce high amounts of recombinant protein. The alcohol oxidase *AOX1* promoter insertion site is commonly used because of its tight regulation and strength^69^. For these reasons, the *AOX1* promoter site was selected as the insertion site in *K. phaffii* without considering closeness to essential genes.

#### Barcoding protocols

Different barcoding methods were designed for each species. For this study, all the selection markers were removed from the final strains, except for *K. phaffii*.

For *E. coli*, *B. subtilis*, *S. albidoflavus*, *S. cerevisiae* and *K. phaffii* vectors containing a restriction site to allow the easy cloning of the barcode sequence by Hi-Fi assembly (NEB) using restriction-linearized vectors were used. The barcode DNA sequences were synthesized as dsDNA fragments (Integrated DNA Technologies) and inserted into the plasmids. The barcoding vectors of *P. putida* were built using the procedures described in^64^ adapted to this microorganism^65^.

Please see Supplementary Table 2 and Supplementary Fig. 2 for a detailed description of the vectors used in this study.

To simplify experiments for this paper, just one DNA sequence was used to barcode each species except for *S. cerevisiae* which each of the two barcoding procedures used a different barcode (Supplementary Table 3). In a real-life scenario, the usage of CellRepo produces different DNA sequence barcodes for each commit (to avoid clashes and ambiguity).

Supplementary Information contains a detailed description of the protocols used to barcode each species and the different growth media used in our studies.

#### Growth curves

For these experiments, all selection markers inserted in the strains were removed (except for *K. phaffii*). All the growth curve experiments were carried out in a CLARIOstar^®^ Plus (BMG Labtech) plate reader using a polystyrene sterile plate, at 300 rpm, using three biological replicates per strain. *E. coli* and *B. subtilis* were grown in LB medium at 37 °C measuring the absorbance at 600 nm. *P. putida* was grown in LB, at 30 °C. *S. cerevisiae* and *K. phaffii* cells were grown at 30 °C in YPD medium in 24-well plates. *S. albidoflavus* plate reader experiment was carried out in TS-agar as previously described in^70^ for *S. coelicolor*.

#### Whole-genome sequencing

The genomic DNA was extracted using: GenElute™ Bacterial Genomic DNA Kit Protocol (Sigma) (*E. coli*, *B. subtilis*, *P. putida* and *S. albidoflavus*) and YeaStar Genomic DNA Kit (Zymo Research) (*S. cerevisiae* and *K. phaffii*).

NGS library was prepared using NEB Next^®^ Ultra™ DNA Library Prep Kit (Cat No. E7370L). Whole-genome sequencing was performed on an Illumina NovaSeq 6000 platform at Novogene (Beijing, China).

The reads were aligned against the reference genome of each species (Supplementary Table 3) using Geneious Prime 2019.2.3 (https://www.geneious.com). First, reads were trimmed using BBDuk (Adapter/Quality Trimming Version 38.37 by Brian Bushnell) and then duplicates were removed using Dedupe (Duplicate Read Remover 38.37 by Brian Bushnell) with default settings in Geneious. The reads were then mapped against the reference genomes using the following settings: Mapper “Geneious”, Sensitivity “Medium/Low/Fast” and selecting the “Find structural variants of any size” option. To annotate SNPs, Geneious integrated algorithm with default settings and a “Minimum variant frequency” value of 0.5 was used.

All the mutations also found in the wild-type strain genome were discarded from the analysis following the next steps:

1. Use the comparison tool in Geneious to remove all the SNPs present in the wild-type strains.
2. Manually curate the rest of SNPs, focusing especially on low coverage and repetitive regions (frequent in both *S. cerevisiae* and *K. phaffii*). SNPs flagged in barcoded strains are not in wild-type strains and vice versa. This is because some detected variants qualify as such in one strain but not in the other due to coverage, quality, etc.

### Barcode survival

The stability of the barcode sequence was tested by growing each species for 10 days under five different growth conditions.

Condition 1: 10 mL of the growth media (LB for *E. coli*, *B. subtilis* and *P. putida*; TSB for *S. albidoflavus*; YPD for *S. cerevisiae* and *K. phaffii*) were inoculated and grown overnight at 200 rpm. Each morning, during the following 10 days, 100 µL of the culture were re-inoculated in 10 mL of fresh media.

Condition 2: 50 mL of the growth media were inoculated and grown at 200 rpm for 10 days.

Condition 3: using a BioXplorer 400 (HEL, London) bioreactor, the cells were grown in 75 mL of growth media with impeller agitation (400 rpm) and filtered air supply (100 mL/min). Cells were grown overnight. After the first night, cells were grown continuously for 10 days at a dilution rate (D) of 0.024 h-1 (minimum possible setting of the system). Antifoam 204 (Sigma A6426) was added to the liquid media before autoclaving at 0.01%.

Condition 4: using the same bioreactor system described in the previous condition cells were grown overnight. After the first night, cells were grown continuously for 10 days at a dilution rate (D) of: 0.3 h-1 for *E. coli*, *B. subtilis* and *P. putida*; 0.2 h-1 for *S. albidoflavus*, *S. cerevisiae* and *K. phaffii*. The dilution rates were inferred from commonly used values for continuous culture^71^ and previously described growth curves.

Condition 5: three colony replicas of the barcoded strains were restreaked on solid media for ten passes.

For conditions 1–4, samples were taken periodically and plated on solid media to ensure no contamination had occurred. On the last day of the experiments, a sample was taken and spread on agar plates of the same growth media. Single colonies were restreaked. For all conditions, the barcode region was amplified by PCR after genomic DNA extraction and sent to Sanger sequencing (Eurofins Genomics). The sequencing results were aligned against the reference barcode sequence for each species.

The genomic DNA of the microbial population of conditions 1–4 were isolated as previously described. Also, as a control experiment that did not go through the 10-day culture period, the gDNA of the glycerol stock used to start the long-term experiments was extracted as well. The barcoded region was amplified by PCR and the amplicon was used for NGS analysis. DNA library preparations, sequencing reactions, and initial bioinformatics analysis were conducted at GENEWIZ, Inc. (South Plainfield, NJ, USA). DNA amplicons with partial adapters were indexed and enriched by limited cycle PCR. The DNA library was validated using TapeStation (Agilent Technologies, Palo Alto, CA, USA), and was quantified using Qubit 2.0 Fluorometer and real-time PCR (Applied Biosystems, Carlsbad, CA, USA). The pooled DNA libraries were loaded on the Illumina instrument according to the manufacturer’s instructions. The samples were sequenced using a 2 × 250 paired-end configuration. Image analysis and base calling were conducted by the Illumina Control Software (HCS) on the Illumina instrument.

Raw Fastq data were first trimmed to remove low-quality data using sickle (https://github.com/najoshi/sickle). PANDAseq (https://github.com/neufeld/pandaseq)^72^ was then used to merge read1 and read2 of each sample. The merged reads of each sample were mapped to the target reference sequence using BWA (http://bio-bwa.sourceforge.net/)^73^. Then variants were detected using GENEWIZ’s in-house script. The primers used for NGS amplicon analysis can be found in Supplementary Table 4.

### Yeast gene drive

Special contingency and sterility measures were taken to perform the gene drive experiments. All the experiments were carried out in a Class 2 safety cabinet. All the surfaces were UV-light and chemically sterilized. All the agar plates were sealed using Parafilm.

pGD-ADE2 was assembled containing homologous regions to *ADE2* gene, *URA3* marker (from BS-Ura3Kl^74^), a sgRNA targeting *ADE2* and a barcode sequence (both chemically synthesized as gBlocks). A modified version of the protocol detailed in^75^ was followed. Briefly, the PCR amplification product of the previous cassette was transformed into BY4741 cells. pCfBf2312 (Cas9) plasmid was transformed afterwards. BY4741-GeneDrive-pCfBf2312 cells were mated with BY4742-pXP622 (used just to select diploids) and plated in SC-Leucine containing G418 (200 μg/mL). Diploid cells were then grown in GNA solid media overnight. Cells were transferred to SPOR plates and sporulated following the protocol described in^76^. Briefly, SPOR plates were incubated at room temperature overnight and 30 °C for 5 days. When tetrads were observed, some cells were scraped from the plate and cell wall digested using Zymolyase solution after incubation at 37 °C for 20 min. Tetrads were dissected using SporePlay+ (Sanger Instruments). Single spores were grown on YPD plates until colony formation. Cells were resuspended in water and 5 µL were transferred to GNA and SC -Uracil plates.

pXP622 was a gift from Nancy DaSilva & Suzanne Sandmeyer (Addgene plasmid # 26849). BS-Ura3Kl was a gift from Zhiping Xie (Addgene plasmid # 69195) (Table 1).

**Table 1:** List of CellRepo-hosted public supplementary data.

Species	Link to CellRepo	QR code
*E. coli*	https://cellrepo.ico2s.org/repositories/59?branch_id=82&locale=en	
*B. subtilis*	https://cellrepo.ico2s.org/repositories/60?branch_id=85&locale=en	
*P. putida*	https://cellrepo.ico2s.org/repositories/61?branch_id=89&locale=en	
*S. albidoflavus*	https://cellrepo.ico2s.org/repositories/62?branch_id=91&locale=en	
*S. cerevisiae*	https://cellrepo.ico2s.org/repositories/65?branch_id=96&locale=en	
*K. phaffii*	https://cellrepo.ico2s.org/repositories/64?branch_id=94&locale=en	

This table provides, for each of the species used in this paper, a URL and a QR code that point to the public repository containing the data associated with each species-specific barcoding protocol.

### Reporting summary

Further information on research design is available in the Nature Research Reporting Summary linked to this article.

## Supplementary information

Supplementary Information

Reporting Summary

## Data Availability

The authors declare that data supporting the findings of this study are available within the paper and its Supplementary Information files. DNA sequencing data are available at NCBI under accession PRJNA797888. In addition, other protocols are publicly available at https://cellrepo.ico2s.org/got as well as at the repositories listed in Table 1.
