# Supplementary material for: Versioning biological cells for trustworthy cell engineering
Source: Nat Commun. 2022 Feb 9;13:765. doi: 10.1038/s41467-022-28350-4 (PMC8828774; doi:10.1038/s41467-022-28350-4)
Supplement: Supplementary file 1 — Supplementary Information [file 41467_2022_28350_MOESM1_ESM.pdf]

Supplementary Information for

Versioning Biological Cells for Trustworthy Cell Engineering

Jonathan Tellechea-Luzardo[1+], Leanne Hobbs[1+], Elena Velázquez[2], Lenka Pelechova[1], Simon Woods[3], Victor de Lorenzo[2] and Natalio Krasnogor[1]*.

Affiliations:

[1]Interdisciplinary Computing and Complex Biosystems (ICOS) Research Group, Newcastle University, Newcastle Upon Tyne NE4 5TG, U.K.

[2]Systems and Synthetic Biology Program, Centro Nacional de Biotecnología (CNB-CSIC), 28049 Madrid, Spain.

[3] Policy Ethics and Life Sciences (PEALS), Newcastle University, Newcastle Upon Tyne NE1 7RU, U.K

[*]Correspondence (Natalio.Krasnogor@newcastle.ac.uk)

[+] These authors contributed equally.

This file includes:

Materials and Methods

Supplementary Figures 1 to 36

Supplementary Tables 1 to 13

Materials and Methods

CellRepo can be accessed here: https://cellrepo.ico2s.org

Detailed protocols

We present here all the step-by-step protocols in "card" format to ease the printing and understanding by CellRepo users.

All the barcoding procedures assume that the barcode has been already cloned into the barcode delivering plasmid and/or that the barcoding cassette has been amplified by PCR.

E. coli barcoding protocols

E. coli λ-Red recombineering		
**Days to completion**	**Reference**	**QR to CellRepo example**
10	[1]	
	**Steps**	
Day 1	1. Start an overnight culture (37 °C) by inoculating LB medium with a single colony.	
Day 2	2. Prepare competent cells following your favourite protocol.<br>3. Transform *E. coli* cells with plasmid pKD46 and plate the cells at 30 °C in LB supplemented with 100 µg/mL ampicillin (or carbenicillin).	
Day 3	4. Start an overnight culture at 30 °C in LB/Amp from a single colony transformant.	
Day 4	5. Next morning refresh the culture (1:100) with LB/Amp and grow the cells until OD_{600} reaches 0.1.<br>6. Add arabinose to a final concentration of 30 mM and grow the cells to an OD_{600} =0.5 (recombination proteins are being expressed at this point).<br>7. Freeze cells on ice for 20 minutes and prepare electrocompetent cells by washing bacteria with ice-cold milli-Q water after spinning aliquots 10 minutes at 5000 rpm in a 4 °C centrifuge.<br>8. After two washes, resuspend cells in the residual water and electroporate with 500 ng of the barcode DNA cassette (coming from the amplification of pEC-Red2-BC) with a Gene Pulser (25 µF, 200 Ω at 1.8 kV).<br>9. After electroporating the cells, add 950 µl of fresh LB without antibiotics to samples and resuspended cultures are grown for 2 hours at 37 °C.<br>10. Plate cells in LB supplemented with chloramphenicol 25 µg/mL.	
Day 5	11. Restreak colonies on LB/CM and grow overnight at 37°C.	
Day 6	12. Perform PCR and sequencing experiments to confirm the insertion of the barcode.<br>13. To remove the antibiotic cassette, the pCP20 plasmid is transformed. Prepare liquid culture of cells containing the barcoding cassette in LB/Cam.	
Day 7	14. Prepare competent cells and transform pCP20 at 30 °C.<br>15. Plate in LB/Cam/Amp	
Day 8	16. After pCP20 transformation, inoculate single colonies in LB/Amp/Cam and grow overnight at 30 °C.	
Day 9	17. Next morning, dilute cells in LB and grow at 30 °C until OD_{600} reaches 0.1<br>18. Swap cells to 42 °C incubator and grow until OD_{600} reaches 0.9.<br>19. Spot 30 µL in LB plate, streak over the plate and incubated at 37 °C.	
Day 10	20. Barcode presence is checked again by PCR and sequencing.<br>21. Restreak single colonies in three different plates (LB, Cam and Amp) to check that the resistance is loss.	

E. coli CRISPR

Days to completion	Reference	QR to CellRepo example
8	[2]	

Steps

Day 1	1. Start an overnight culture (37 °C) by inoculating LB medium from a single colony.
Day 2	2. Prepare competent cells following your favorite protocol.<br>3. Transform *E. coli* cells with plasmid pREDCas9 and plate the cells at 30 °C in LB supplemented with 50 µg/mL spectinomycin.
Day 3	4. Start an overnight culture at 30 °C in LB/Spec from a single colony.
Day 4	5. Next morning refresh the culture with LB/Spec and grow the cells until OD600 reaches 0.1.<br>6. Add IPTG to a final concentration of 2 mM and grow the cells to an OD600 =0.6 (recombination proteins are being expressed at this point).<br>7. Frost cells on ice for 20 minutes and electrocompetent cells are then prepared by washing bacteria with ice-cold milliQ water after spinning aliquots 10 minutes at 5000 rpm in a 4 °C centrifuge. (Heat shock transformation also could be performed)<br>8. After two washes, resuspend cells in the residual milliQ water and electroporate with pEC-CRISPR2-BC.<br>9. Add 950 µl of fresh LB without antibiotics and resuspend cells. Incubate cells 1 hour at 30 °C.<br>10. Then, spread 100 µL of bacterial culture on LB plates supplemented with Spec (50 µg/mL) /Amp (100 µg/mL).
Day 5	11. Check colonies for barcode presence by colony-PCR.<br>12. Inoculate a positive clone in 2 mL of LB/Spec/Ara (30 mM) (sgRNA targeting pUC origin in pEC-CRISPR2-BC is expressed).<br>13. Grow for 4-6 hours.<br>14. Plate on LB/Spec.
Day 6	15. Check some colonies for Ampicillin resistance by restreaking them on LB/Amp.
Day 7	16. Take a sensitive clone and restreak it on LB plates at 37°C.
Day 8	17. Check again by colony PCR the presence of the barcode and restreak on LB/Spec and LB/Amp plates to double check plasmid curing.

Note: Different sgRNA sequences were used in different experiments (obtained using [3]):

- Final sgRNA1: 5’ - promoter – ATTCCGCGTAAGTATCGCGG– scaffold – terminator 3’
- Final sgRNA2: 5’ - promoter – CGTACAAAAGTACGTGAGGA– scaffold – terminator 3’

B. subtilis barcoding protocols

The toxin-antibiotic cassette assembly was achieved by HiFi assembly (NEB) of all the parts. The final product was PCR amplified and transformed into 168 cells.

B. subtilis **mazF toxin mediated barcoding**		
**Days to completion**	**Reference**	**QR to CellRepo example**
6	[4]	
**Steps**		
Day 1	1. Inoculate *B. subtilis* cells from a single colony overnight at 37 °C in minimal medium (MM: 10 ml SMM basic salts, 125 µl 40 % (w/v) glucose, 100 µl 2 % (w/v) tryptophan, 60 µl 1 M Mg2SO4*7H2O, 10 µl 20 % (w/v) casamino acids, 5 µl 2.2 mg/ml ferric ammonium citrate)	
Day 2	2. In the morning, dilute cells 1:100 in MM and incubate for 3 h at 37 °C.<br>3. Dilute 1:2 in SM (SM: 10 ml SMM basic salts, 125 µl 40 % (w/v) glucose, 60 µl 1 M Mg2SO4*7H2O). Incubate 2 h at 37 °C.<br>4. Mix 400 µL of cells with 1µg of barcode DNA cassette coming from the PCR amplification of the HiFi assembly of all the parts. Incubate 1 h at 37 °C.<br>5. Plate cells on NA supplemented Zeocin (20 µg/mL) plates. Incubate overnight at 37 °C.	
Day 3	6. Check integration by colony PCR.<br>7. Incubate overnight one positive clone at 37 °C in LB/0.4% (w/v) glucose/zeocin.	
Day 4	8. Dilute the culture to OD600=0.1 in fresh LB/O.4% (w/v) glucose without antibiotics and grow to OD600=0.4.<br>9. Add 1 % (w/v) xylose. Incubate 8 h at 37 °C.	
Day 5	10. Plate cells on NA supplemented with 1 % (w/v) xylose.	
Day 6	11. Restreak individual colonies on NA and NA/Zeocin and colony-PCR to test for cassette removal.	

B. subtilis Cre-Lox

Days to completion	Reference	QR to CellRepo example
6	5	

Steps	
Day 1	1. Start an overnight culture 37 °C in minimal medium.
Day 2	2. Dilute cells 1:10 in MM and grown for 3 h at 37 °C. Meanwhile, SM is prepared and prewarmed at 37 °C.<br>3. Dilute in 1:2 SM and make competent cells with a further 2 h incubation period at 37 °C.<br>4. 400 µL cell aliquots are mixed with 1 µg recombinant DNA PCR (coming from the amplification of pBS-CreLox-BC). Incubate 1 h at 37 °C.<br>5. Cells are spun down, concentrated and plated on NA/Zeocin (20 µg/mL) and incubated at 37°C.
Day 3	6. Colonies are tested for the integration of the recombinant DNA by PCR and sequencing.<br>7. Grow a positive clone overnight at 37 °C in MM.
Day 4	8. Dilute cells 1:10 in MM and grown for 3h at 37°C. Meanwhile, SM is prepared and prewarmed at 37 °C.<br>9. The culture is diluted 1:2 in SM and cells are made competent with a further 3 h incubation period at 37 °C.<br>10. 400 µl cell aliquots are mixed with 100 ng pDR244.<br>11. Cells are spun down, concentrated and plated on LB supplemented with spectinomycin (100 µg/mL) and incubated at 30 °C.
Day 5	12. Check selection cassette removal by colony PCR.<br>13. Positive clones are plated on NA/Zeocin (to check ABR loss) and on LB at 37 °C to cure pDR244.
Day 6	14. Check pDR244 curation by plating cells on LB/Spec.

B. subtilis CRISPR

Days to completion	Reference	QR to CellRepo example
4	[6]	

Steps	
Day 1	1. Start an overnight culture 37 °C in minimal medium.
Day 2	2. Dilute cells 1:10 in MM and grown for 3 h at 37 °C. Meanwhile, starvation medium is prepared and prewarmed at 37 °C.<br>3. Dilute in 1:2 SM and make competent cells with a further 2 h incubation period at 37 °C.<br>4. Mix 400 µl cell with 400 ng pBS-CRISPR-BC. Spin down cells and plate on LB supplemented with 5 µg/mL kanamycin and 0.2% mannose and incubate at 30 °C for 1 h.
Day 3	5. Check barcode presence by colony-PCR.<br>6. Cure positive clones from pBS-CRISPR-BC by restreaking them at 37 °C in LB plates.
Day 4	7. Store kanamycin sensitive clones as barcoded.

Final sgRNA: 5' - promoter – GGAAAAGAGTATATTAGATA – scaffold – terminator 3'.

sgRNA sequences obtained using [3].

P. putida barcoding protocol

The barcoding protocol using TargeTron was adapted from *E. coli*[8] to *P. putida*[9]. The barcode sequence was synthesized by PCR with two overlapping oligonucleotides. Afterwards, this PCR fragment was directly cloned into MluI-digested pSEVA6511-GIIi by Gibson Assembly to generate pSEVA6511-GIIi-BC. The generation of pSEVA231-C-94a is explained elsewhere[10]. Details on how the TargeTron system works as a barcode deliverer in *P. putida* can be found in Supplementary Fig. 3. After generating these two plasmids, the barcoding protocol was carried out as follows:

***P. putida* CRISPR/targetron**		
**Days to completion**	**Reference**	**QR to CellRepo example**
7	9	
**Steps**		
Day 1	1. Start an overnight culture (30° C) by inoculating LB medium from a single colony.	
Day 2	2. Transform plasmid pSEVA421-Cas9tr into *P. putida* strain either by electroporation[11] or conjugation[12]:<br>o Electroporation: Briefly, wash an overnight culture with 300mM sucrose at room temperature four times. At the end, resuspend pellet in 400 µL of sucrose and separate in 100 µL aliquots. After adding 100 ng of pSEVA421-Cas9tr to one aliquot, carry out the electrical shock (program in Gene Pulser from Bio-Rad: 2.5 kV, 25 µF, 200 Ω.) and plate on LB supplemented with streptomycin (100 µg/mL).<br>o Conjugation: Tri-parental mating can be performed for the mobilization of pSEVA plasmids from *E. coli* to *P. putida* strains. Briefly, take 1 mL from overnight cultures of donor (*E. coli* strain bearing pSEVA421-Cas9tr), helper (in our case, *E. coli* HB101 with plasmid pRK600) and recipient *P. putida* strain. Spin down cells and wash once with 10mM MgSO4. Mix 100 µL from each washed culture and spin down cells to eliminate the supernatant. Finally, resuspend the final pellet in 20 µL of $MgSO_4$ and spot on a LB agar plate. When dry, incubate for at least 4h at 30°C and then, recuperate cells, resuspend them on $MgSO_4$ and plate serial dilution on M9 minimal media supplemented with sodium citrate at 0.2% (w/v) and streptomycin.	
Day 3	3. Select single colonies resistant to streptomycin and start a new culture.	
Day 4	4. Transform plasmid pSEVA6511-GIIi-BC by following one of the same two procedures described above.	
Day 5	5. Select single colonies resistant to streptomycin and gentamycin (15 µg/mL)<br>6. Start a new culture (20 mL of LB supplemented with streptomycin and gentamycin is recommended)	
Day 6	7. Induce the overnight culture with 1mM cyclohexanone for 4h at 30° C<br>8. Make competent cells by following the procedure explained before Electroporate 100 ng of pSEVA231-C-94a into one aliquot of competent cells and recover the culture for 2 h in LB supplemented with streptomycin.<br>9. Plate the cells on LB plates supplemented with streptomycin and kanamycin (50 µg/mL).	
Day 7	10. Check by PCR the presence of the barcode at the correct locus and start giving passages with no antibiotic selection to cure plasmids.	

S. albidoflavus barcoding protocol

pCRISPomyces-2 vector was used to engineer *S. albidoflavus* J1074. Using the vector construction protocol detailed in[13], pSA-CRISPR vector was built (testing two different sgRNA sequences). By Hi-Fi assembly, the barcode sequence was cloned. To transform this vector into *S. albidoflavus* J1074, the protocol detailed in [14] was followed. pCRISPomyces-2 was a gift from Huimin Zhao (Addgene plasmid # 61737).

Note: Two sgRNA sequences used (obtained using [5]):

⇒ sgRNA1: 5' - promoter – TCATCGTTCTCAATACACCG– scaffold – terminator 3'
⇒ sgRNA2: 5' - promoter – TGCAACCTCCGTGATCATTC– scaffold – terminator 3'

***S. albidoflavus* CRISPR**		
**Days to completion**	**Reference**	**QR to CellRepo example**
8	13,14	
**Steps**		
Day 1	1. Start an overnight culture of *E. coli* ET12567 (conjugative strain used to transform by the plasmid into Streptomyces species) at 37°C in LB supplemented with Chloramphenicol and Kanamycin.	
Day 2	2. Prepare competent cells of *E. coli* ET12567.<br>3. Transform using your favourite protocol with pSA-CRISPR-BC.<br>4. Plate in LB supplemented with chloramphenicol (25 µg/mL), kanamycin (50 µg/mL) and apramycin (50 µg/mL).	
Day 3	5. Pick one colony and inoculate 5 mL of LB supplemented with Chloramphenicol, Kanamycin and Apramycin.	
Day 4	6. Pellet down cells.<br>7. Wash cells 3 times with 1 mL 2xYT media.<br>8. Heat shock ~108 *S. albidoflavus* spores in 100 uL of 2xYT at 50°C for 10 min.<br>9. Use the 100uL spores to resuspend the *E. coli* pellet.<br>10. Plate on MS-agar supplemented with 20mM MgCl2.<br>11. Incubate overnight at 30°C.	
Day 5	12. Dissolve Apramycin and Nalidixic Acid (which does not act against *S. albidoflavus*) in 1mL H2O.<br>13. Overlay the antibiotic mixture on the plates. Let them dry.<br>14. Incubate at 30°C for 1 week.	
Day 6	15. Restreak 10 *S. albidoflavus* colonies on MS/Nal at 37°C to cure the plasmids.<br>16. Grow until colonies appear.<br>17. Repeat three times.	
Day 7	18. Restreak on MS-agar/Apra to check plasmid curing.	
Day 8	19. Extract genomic DNA of cells using Sigma's Genomic extraction Kit.<br>20. Check barcode presence by PCR.	

S. cerevisiae barcoding protocols

S. cerevisiae Cre-Lox		
**Days to completion**	**Reference**	**QR to CellRepo example**
8	[15]	

Steps

Day 1	1. Start an overnight culture of *S. cerevisiae* cells in YPD.
Day 2	2. Inoculate 5mL of YPD with 500uL of culture at 30°C.<br>3. Incubate until OD=0.2-0.3.<br>4. Prepare competent cells by the LiAc method.<br>5. Transform with the PCR product coming from the cassette amplification of pSC-CreLox-BC ((both KanMX and *URA3* markers were tested)<br>6. Incubate in YPD for 2 hours (if the selected marker is *URA3*, this step can be skipped).<br>7. Plate on YPD supplemented with 200 µg/mL of G418 (or SC -uracil) at 30°C.
Day 3	8. Wait until colonies appear.<br>9. Check by PCR barcode presence.<br>10. Inoculate one positive clone in YPD.
Day 4	11. Repeat protocol of Day 1 to transform pBF3060_NatMX (cre recombinase expression).<br>12. Plate on YPD supplemented with 100 µg/mL Neurothreocin.<br>13. Plate at 30°C.
Day 5	14. Inoculate 2 mL of appropriate media to maintain selection but with 2% Raffinose /0.1% Glucose as the carbon source.<br>15. Grow overnight at 30°C.
Day 6	16. Make a 1/10 dilution using the appropriate selection media supplemented with 2% Galactose and 0.1% Glucose (this induces the expression of CreA).<br>17. Plate cells in YPD/Neurothreocin.
Day 7	18. Check selection cassette removal by colony PCR<br>19. Inoculate 2 mL of YPD without antibiotic with a positive clone (this step may need to be repeated 2 or 3 days to allow the plasmid curation).<br>20. Grow overnight at 30°C.
Day 8	21. Plate on YPD after dilution looking for single colonies.<br>22. Check the single colonies for Neurothreocin sensitivity (pSC-Crelox-BC curation).

pBF3060 was a gift from Nancy DaSilva & Suzanne Sandmeyer (Addgene plasmid # 26850).

S. cerevisiae CRISPR

Days to completion	Reference	QR to CellRepo example
6	16	

Steps	
Day 1	1. Start an overnight culture of *S. cerevisiae* cells in YPD.
Day 2	2. Inoculate 5mL of YPD with 500uL of culture at 30°C.<br>3. Incubate until OD=0.2-0.3<br>4. Prepare competent cells by the LiAc method.<br>5. Transform with pCfBf2312 (Cas9).<br>6. Incubate in YPD for 2 hours.<br>7. Plate on YPD supplemented with 200 µg/mL G418 at 30°C.
Day 3	8. Wait until colonies appear.<br>9. Inoculate a positive clone into YPD/G418 at 30°C.
Day 4	10. Repeat protocol of Day 1 to co-transform pCfBf3020 (gRNA) and the PCR product coming from pSC-CRISPR-BC.<br>11. Incubate in YPD for 2 hours.<br>12. Plate on YPD supplemented with Neurothreocin (100 µg/mL) and G418 (200 µg/mL) at 30°C.
Day 5	13. Wait until colonies appear.<br>14. Check by PCR barcode presence<br>15. Inoculate a positive clone in YPD without antibiotic (this step may need to be repeated 2 or 3 days to allow the plasmids curation).
Day 6	16. Plate on YPD after dilution looking for single colonies.<br>17. Test Neurothreocin and G418 sensitive colonies.

In short, the procedure is based on two plasmids and a PCR repair template.

pCfBf2312, pCfBf2899 and pCfBf3020 were a gift from Irina Borodina (Addgene plasmid # 78231, 73271 and 73282 respectively).

sgRNA: 5’ - promoter – CTCTCGAAGTGGTCACGTGC– scaffold – terminator 3’.

K. phaffi barcoding protocol

Adapted from “Thermo Fisher Scientific’s pPICZ A, B, and C transformation protocol (Manual part no. 25-0148)”. A version of pPICZ vector, pICXNH3[17] was used to create our vector. The barcode sequence was inserted after the *AOX1* terminator sequence. The vector allows the integration of any desired protein after the *AOX1* promoter. SacI was used to linearize the plasmid. pICXNH3 was a gift from Raimund Dutzler & Eric Geertsma (Addgene plasmid # 49020).

***K. phaffii* barcoding**		
**Days to completion**	**Reference**	**QR to CellRepo example**
5	“Thermo Fisher Scientific’s pPICZ A, B, and C transformation protocol (Manual part no. 25-0148)”	

**Steps**	
Day 1	1. Digest ~5–10 µg of barcoded plasmid DNA with SacI<br>2. Check linearization in agarose gel<br>3. Column purify the digestion reaction<br>4. Grow 5 mL of Pichia pastoris strain in YPD at 30°C overnight.
Day 2	5. Inoculate 50 mL of fresh medium with the overnight culture.<br>6. Grow overnight again to an OD600 = 1.3–1.5.
Day 3	7. Centrifuge the cells at 1,500 × g for 5 minutes at 4°C. Resuspend the pellet with 500 ml of ice-cold, sterile water.<br>8. Centrifuge again, then resuspend the pellet with 250 ml of ice cold, sterile water.<br>9. Centrifuge again, then resuspend the pellet in 20 ml of ice-cold 1 M sorbitol.<br>10. Centrifuge again, then resuspend the pellet in 1 ml of ice-cold 1 M sorbitol for a final volume of approximately 1.5 ml. Keep the cells on ice and use that day. Do not store cells.<br>11. Mix 80 µL of the cells from Step 6 (previous page) with 5–10 µg of linearized DNA (in 5–10 µL sterile water) and transfer them to an ice-cold 0.2 cm electroporation cuvette.<br>12. Incubate the cuvette with the cells on ice for 5 minutes.<br>13. Pulse the cells using the manufacturer’s instructions for *Saccharomyces cerevisiae*.<br>14. Immediately add 1 ml of ice-cold 1 M sorbitol to the cuvette. Transfer the cuvette contents to a sterile 15-ml tube and incubate at 30°C without shaking for 1 to 2 hours.<br>15. Spread 10, 25, 50, 100, and 200 µl each on separate, labelled YPDS plates containing 100 µg/ml Zeocin. Plating at low cell densities favours efficient Zeocin™ selection.<br>16. Incubate plates from 3–10 days at 30°C until colonies form.
Day 4	17. Pick 10–20 colonies and purify (streak for single colonies) on fresh YPD or YPDS plates containing 100 µg/ml Zeocin.
Day 5	18. Colony PCR to check barcode presence.

Recipes of media used in this study

LB (1 L):

- 10 g tryptone
- 10 g NaCl
- 5 g yeast extract
- Up to 1 L distilled water

SMM (1 L):

- 2 g Ammonium sulphate
- 14 g Dipotassium hydrogen phosphate
- 6 g potassium dihydrogen phosphate
- 1 g trisodioum citrate dihydrate
- 0.2 g magnesium sulphate heptahydrate
- Up to 1 L distilled water

B. subtilis MM (10 mL):

- 10 mL SMM basic salts
- 125 µL 40% (w/v) glucose
- 100 µL 2% (w/v) tryptophan
- 60 µL 1M $Mg2SO_4*7H_2O$
- 10 µL 20% (w/v) casamino acids
- 5 µL 2.2mg/ml ferric ammonium citrate.

B. subtilis starvation media (10 mL):

- 10 mL SMM basic salts
- 125 µL 40% (w/v) glucose
- 60 µL 1M $Mg2SO_4*7H_2O$

M9 minimal medium recipe (1L):

- 6 g Na_2HPO_4
- 3 g KH_2PO_4
- 0.5 g NH_4Cl
- 0.5 g NaCl
- 0.2 g $MgSO_4 \cdot 7H_2O$

NA plates (100 mL):

- 2.8 g oxoid nutrient agar
- Up to 100 mL distilled water

TSB (1 L):

- 30 g Tryptic Soy Broth powder
- Up to 1 L distilled water

2xYT (1 L)

- 16 g tryptone
- 10 g yeast extract
- 5 g NaCl
- Up to 1 L distilled water.

MS plates (1 L):

- 20 g mannitol
- 20 g soy flour
- 20 g agar
- Up to 1 L distilled water

YPD (1 L):

- 20 g peptone
- 10 g yeast extract
- Up to 1 L distilled water
- Add glucose to 2% final concentration after autoclaving

SC dropout plates (100 mL) (compounds from Formedium):

- 690 mg Nitrogen base without amino acids
- 162.2 mg of Leucine dropout or 192.6 mg of Uracil dropout mixture
- 2.4 g agar
- Up to 100 mL

SC dropout plates without ammonium sulphate (100 mL) (compounds from Formedium):

- 190 mg Nitrogen base without amino acids and without ammonium sulphate
- 162.2 mg of Leucine dropout or 192.6 mg of Uracil dropout mixture
- 2.4 g agar
- Up to 100 mL

GNA plates (100 mL):

- 5 g D-glucose
- 3 g Nutrient broth
- 1 g yeast extract
- 2.4 g agar
- Up to 100 mL distilled water

SPOR plates (100 mL):

- 1 g potassium acetate
- 100 mg yeast extract
- 50 mg glucose
- 2.4 g agar
- Up to 100 mL distilled water

Supplementary Figures

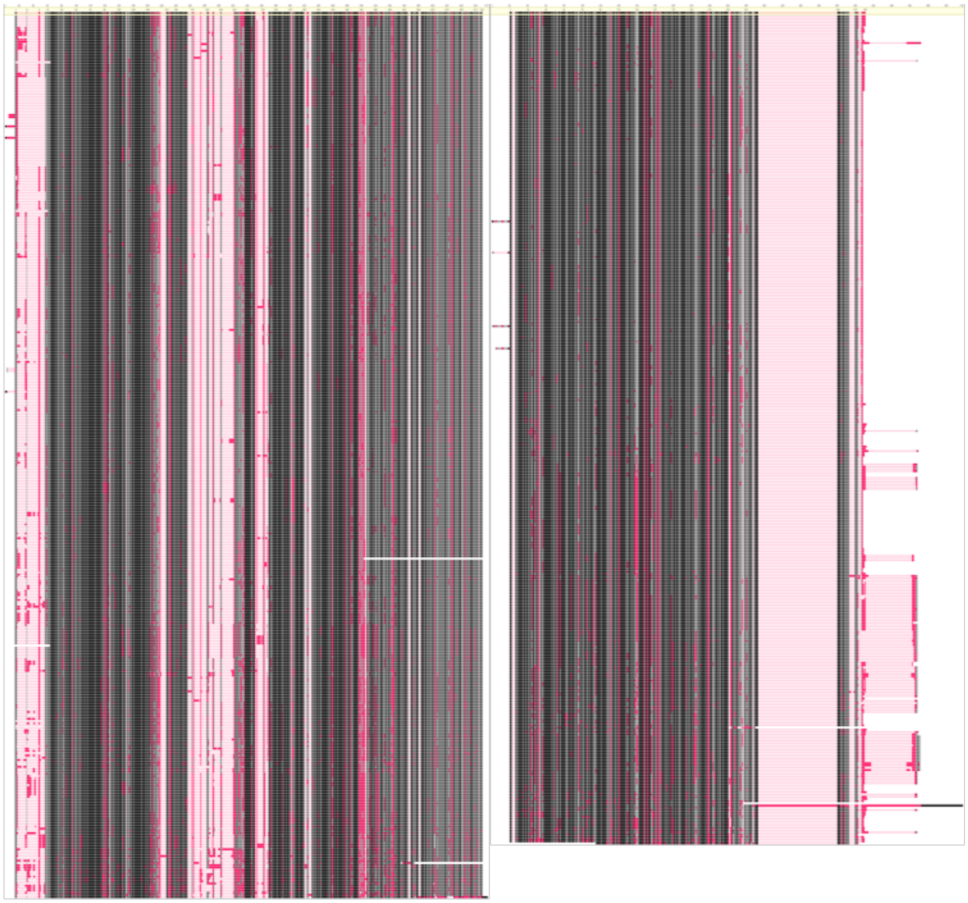

	AGI90205.1	AGI90207.1
Alignment length	677	518
Sequences	387	373
Identical Sites	163 (24.4%)	91 (19.6%)
Pairwise Identity	83.4%	85.3%

Supplementary Fig. 1.

Conservation pattern of the putative essential gene pair AGI90205.1 (IMP cyclohydrolase / Phosphoribosylaminoimidazolecarboxamide formyltransferase) and AGI90207.1 (Bifunctional protein FolD) (encoded by loci XNR_3870 and XNR_3872 respectively). Both protein sequences were aligned against the classified Streptomyces database using BLAST. The hits were aligned using MAFFT included in Geneious Software package. Black colour indicates a higher similarity. Pink colour indicates a lower similarity. Spaces indicate alignment gaps. The alignment statistics were calculated by Geneious.

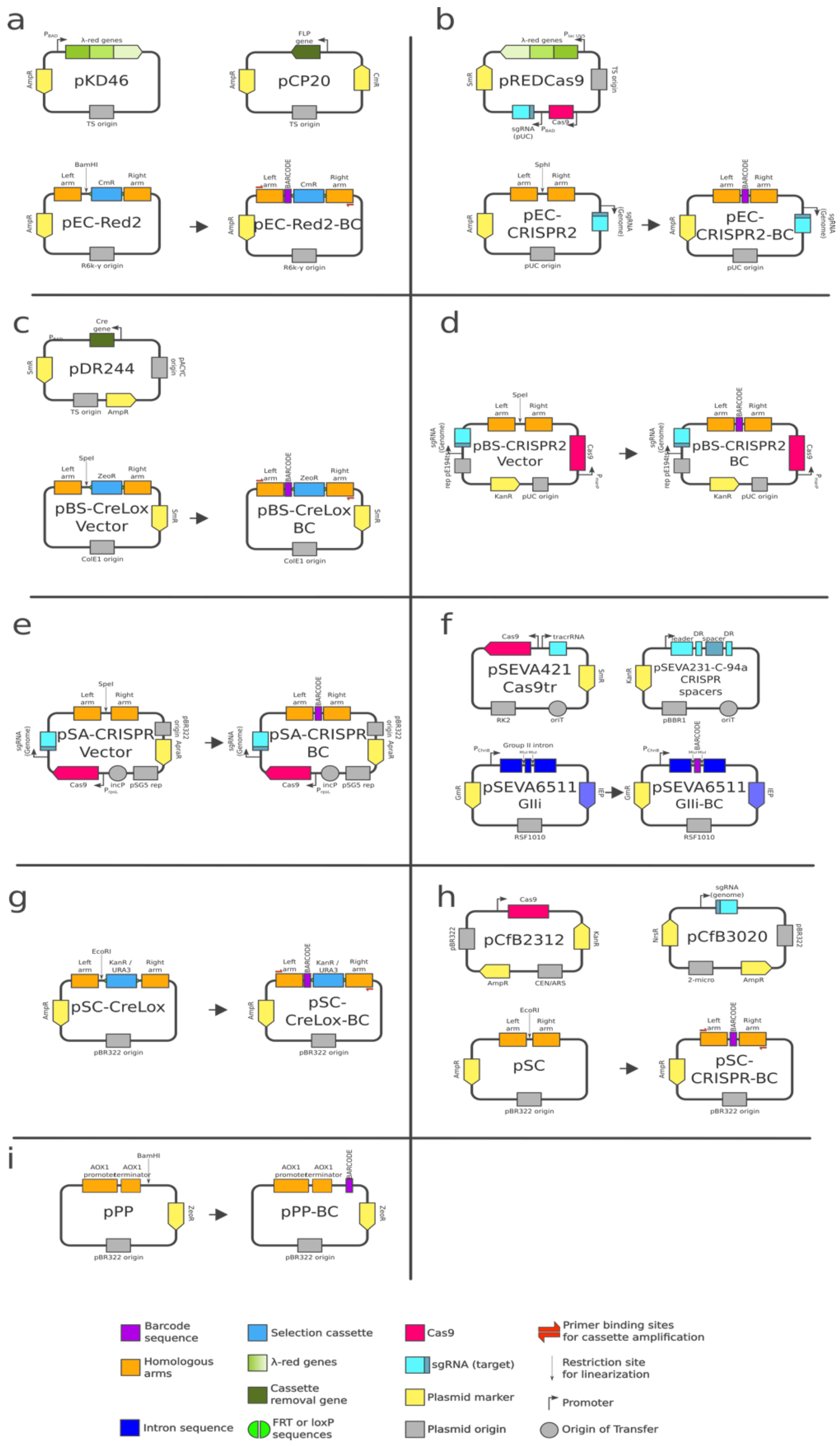

Supplementary Fig. 2.

Maps of the barcoding plasmids used in this study. a) *E. coli* lambda-red. b) *E. coli* CRISPR. c) *B. subtilis* Cre-Lox. d) *B. subtilis* CRISPR. e) *S. albidoflavus* CRISPR. f) *P. putida* CRISPR/targetron. g) *S. cerevisiae* Cre-Lox. h) *S. cerevisiae* CRISPR. i) *K. phaffii AOX1* insertion.

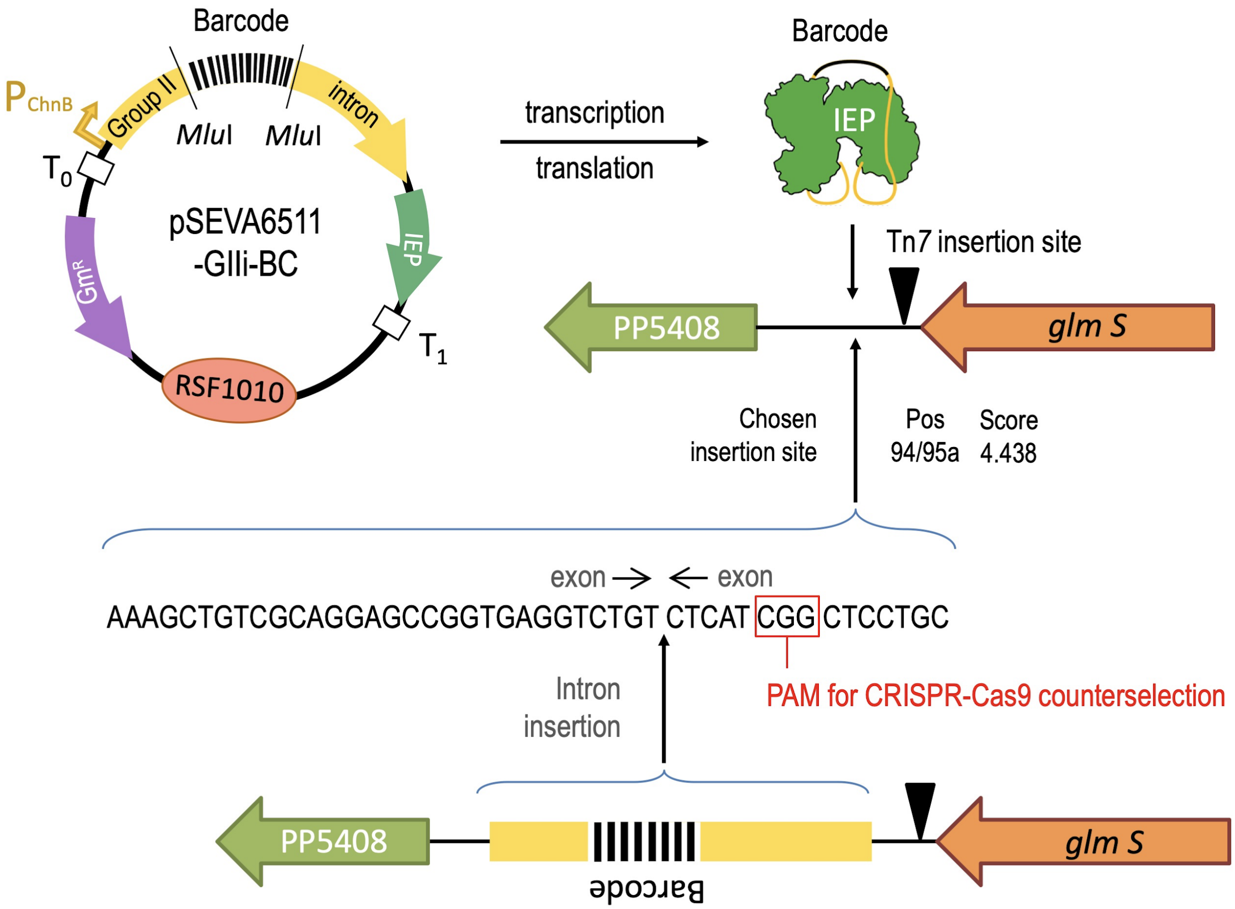

Supplementary Fig. 3.

Insertion of a barcode in a permissive site of the genome of *Pseudomonas putida*. Once committed, the barcode was assembled as part of the Group II intron sequence of plasmid pSEVA6511-GIIi-BC, which co-transcribes the corresponding RNA along with an intron-encoded protein (IEP) with reverse transcriptase activity. Upon induction of the expression system with cyclohexanone, a ribonucleoprotein (RNP) complex is formed that contains the excised intron RNA and IEP. After RNA splicing, the group II intron RNP recognizes DNA target sequences for intron insertion by using both the IEP and base pairing of the intron RNA. In our case the site of insertion is chosen in the proximity of the Tn7 insertion site and is predicted to occur with the target identification algorithm in the site indicated. Note also the PAM site exploited for counterselection of the non-inserted, wild-type region with the CRISPR/Cas9 system explained in the text.

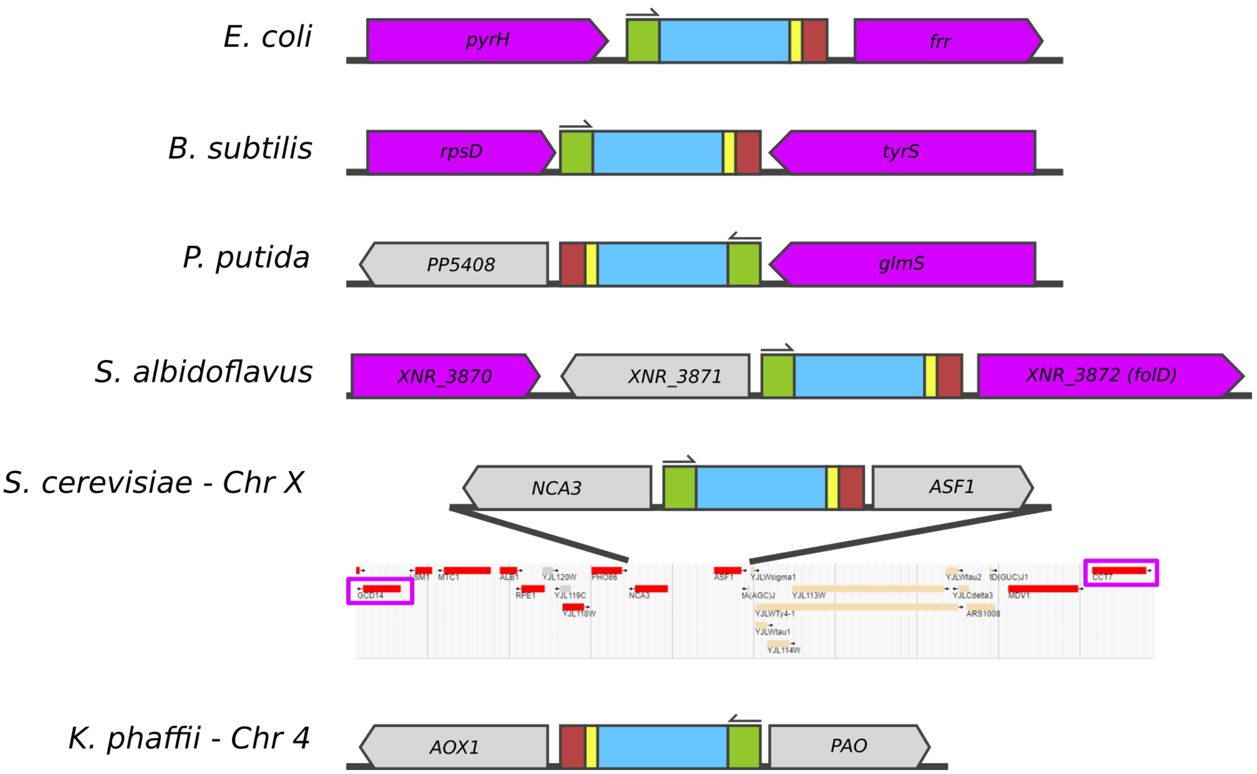

Supplementary Fig. 4.

Barcoding locations used in this study. Purple colour indicates essential genes. Universal sequencing primer site (green), barcode sequence (blue), synchronisation sequence (yellow) and checksum (red). *S. cerevisiae* genomic map was obtained from https://www.yeastgenome.org/

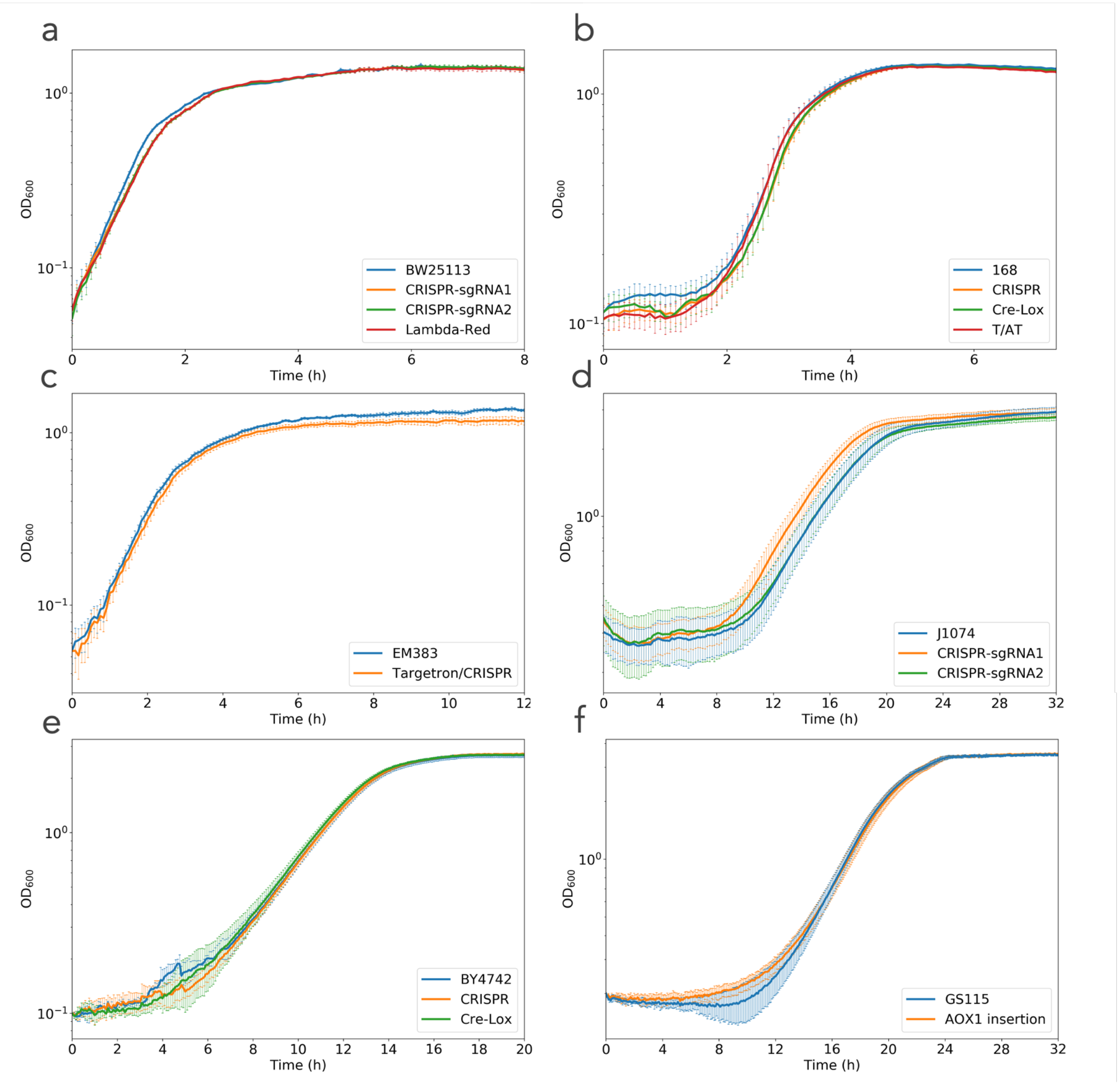

Supplementary Fig. 5.

Growth profile comparison between wild-type strains and barcoded strains. Wild-type strains are shown in blue. a) *E. coli.* b) *B. subtilis*. c) *P. putida*. d) *S. albidoflavus*. e) *S. cerevisiae*. f) *K. phaffii.* Note that the variation in *S. albidoflavus* graph can be explained due to cells grown on solid tryptic soy agar. n=3 biologically independent colonies were used for each control and barcoded strain. Data are presented as mean values +/- standard deviation.

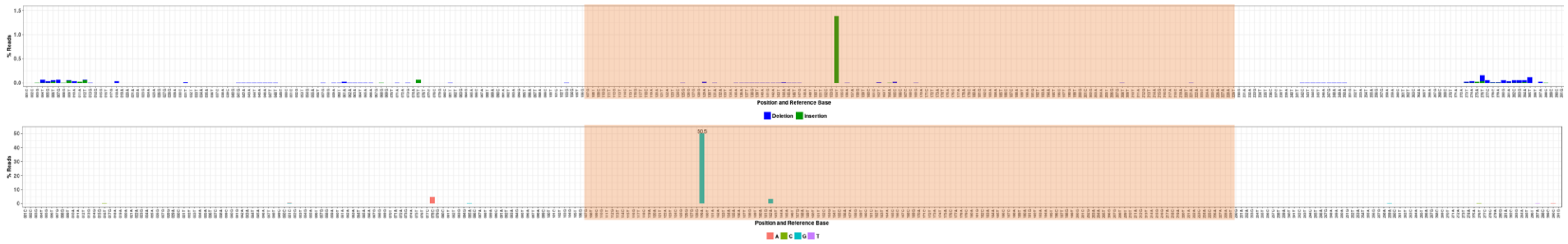

Supplementary Fig. 6.

E. coli amplicon NGS. Glycerol control. Barcode sequence indicated by the orange region. Upper graph: percentage of reads showing a specific deletion/insertion. Lower graph: percentage of reads showing a specific base pair change. ~17000 read depth.

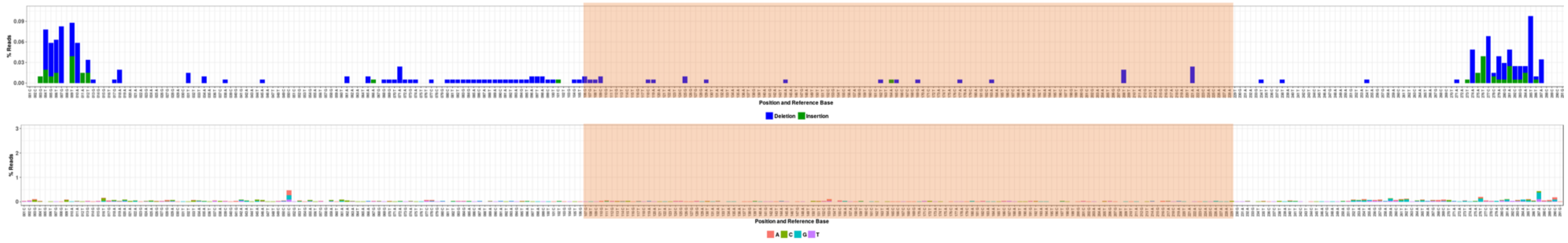

Supplementary Fig. 7.

E. coli amplicon NGS. Condition 1. Barcode sequence indicated by the orange region. Upper graph: percentage of reads showing a specific deletion/insertion. Lower graph: percentage of reads showing a specific base pair change. ~19500 read depth.

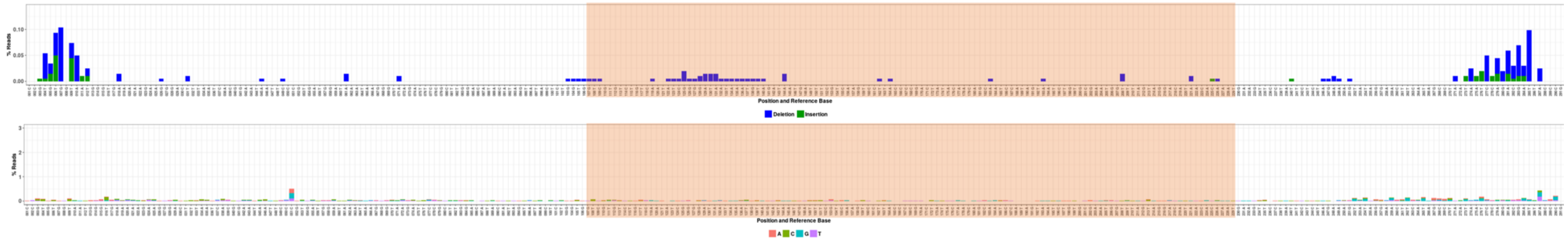

Supplementary Fig. 8.

E. coli amplicon NGS. Condition 2. Barcode sequence indicated by the orange region. Upper graph: percentage of reads showing a specific deletion/insertion. Lower graph: percentage of reads showing a specific base pair change. ~19200 read depth.

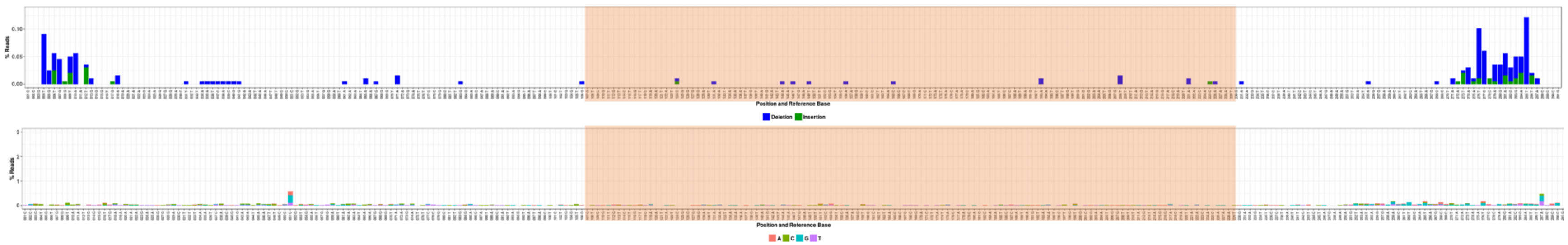

Supplementary Fig. 9.

E. coli amplicon NGS. Condition 3. Barcode sequence indicated by the orange region. Upper graph: percentage of reads showing a specific deletion/insertion. Lower graph: percentage of reads showing a specific base pair change. ~18700 read depth.

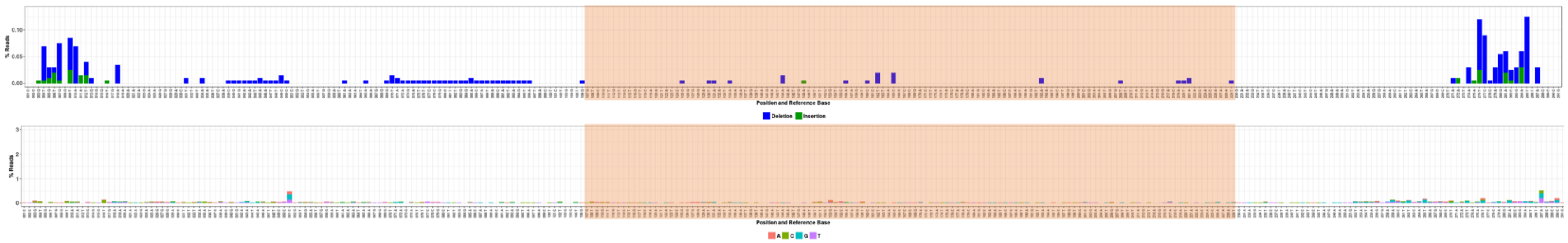

Supplementary Fig. 10.

E. coli amplicon NGS. Condition 4. Barcode sequence indicated by the orange region. Upper graph: percentage of reads showing a specific deletion/insertion. Lower graph: percentage of reads showing a specific base pair change. ~19200 read depth.

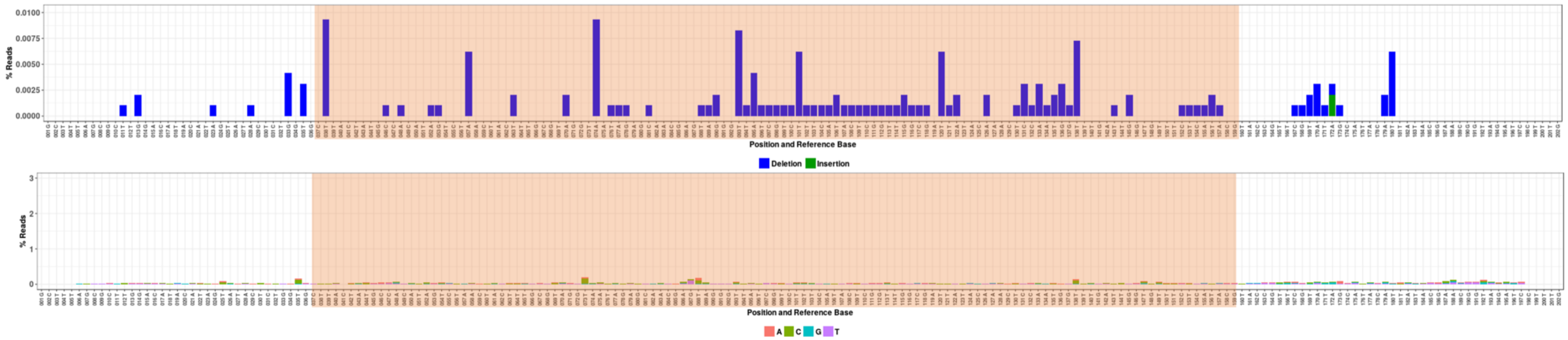

Supplementary Fig. 11.

B. subtilis amplicon NGS. Glycerol control. Barcode sequence indicated by the orange region. Upper graph: percentage of reads showing a specific deletion/insertion. Lower graph: percentage of reads showing a specific base pair change. ~95500 read depth.

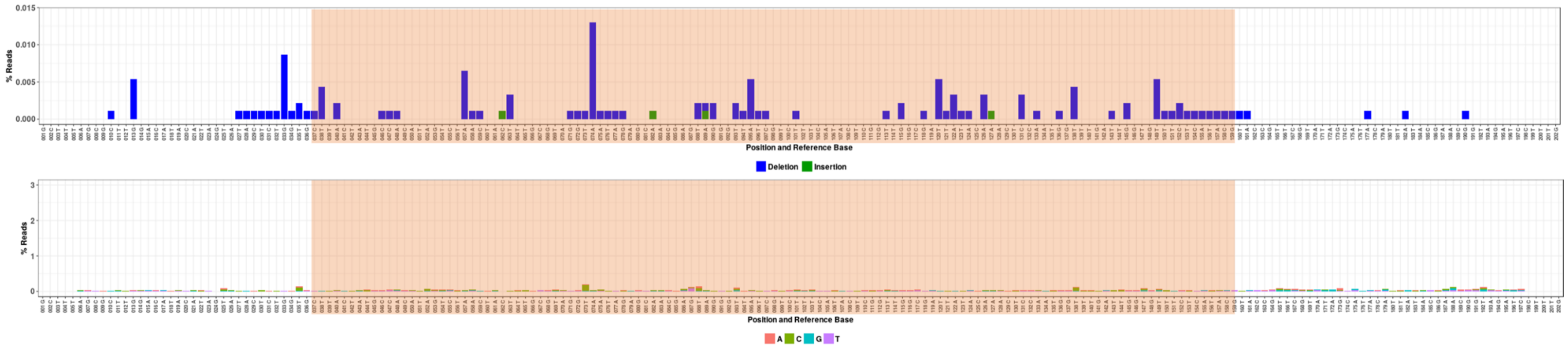

Supplementary Fig. 12.

B. subtilis amplicon NGS. Condition 1. Barcode sequence indicated by the orange region. Upper graph: percentage of reads showing a specific deletion/insertion. Lower graph: percentage of reads showing a specific base pair change. ~91400 read depth.

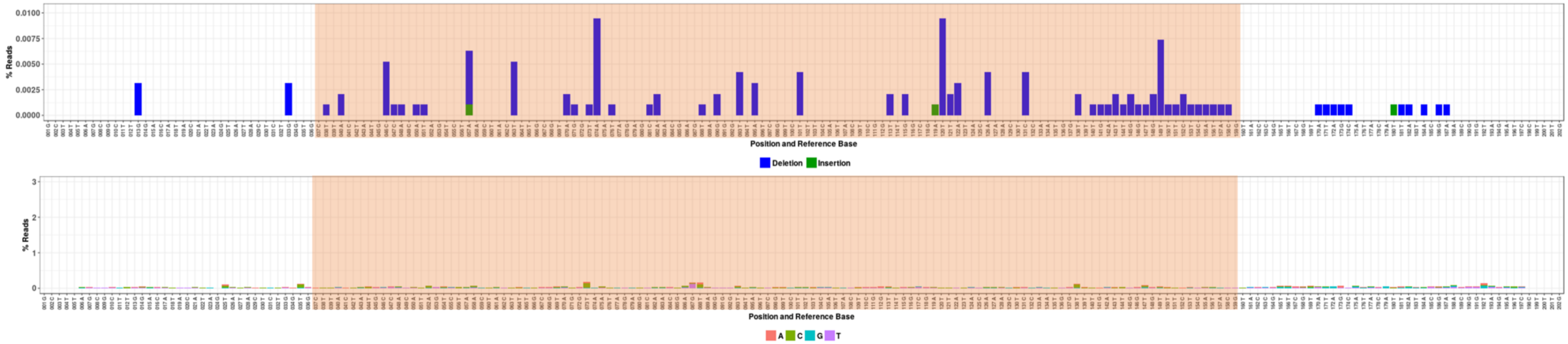

Supplementary Fig. 13.

B. subtilis amplicon NGS. Condition 2. Barcode sequence indicated by the orange region. Upper graph: percentage of reads showing a specific deletion/insertion. Lower graph: percentage of reads showing a specific base pair change. ~94300 read depth.

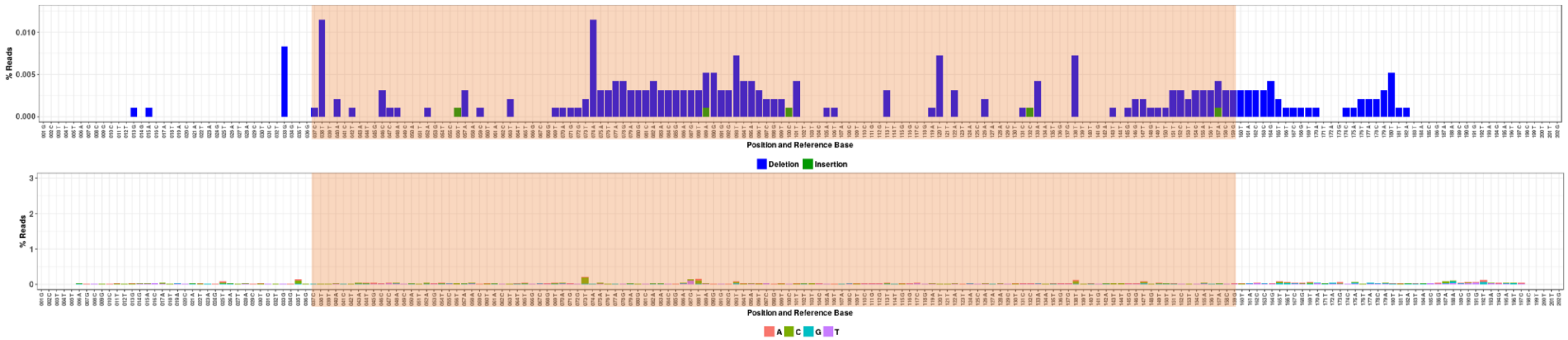

Supplementary Fig. 14.

B. subtilis amplicon NGS. Condition 3. Barcode sequence indicated by the orange region. Upper graph: percentage of reads showing a specific deletion/insertion. Lower graph: percentage of reads showing a specific base pair change. ~95000 read depth.

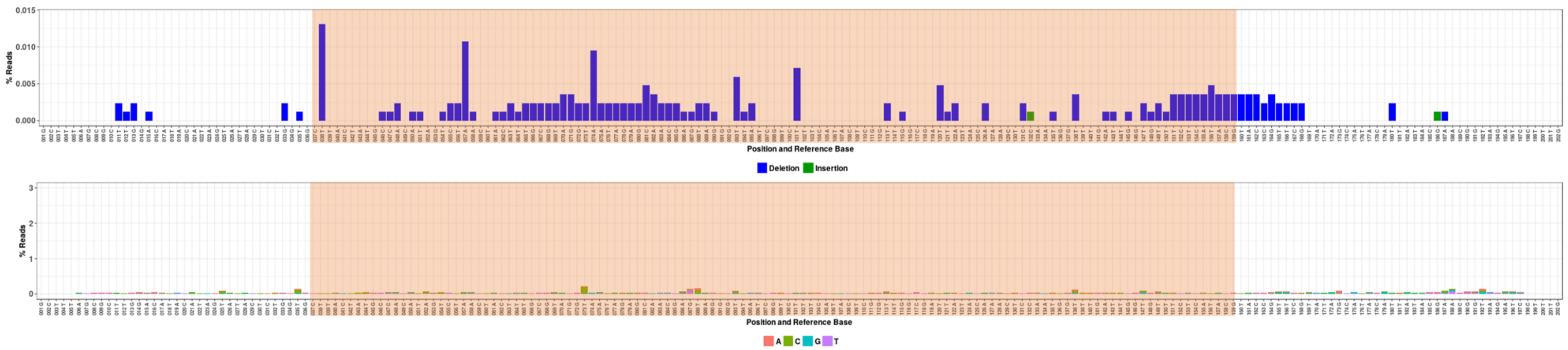

Supplementary Fig. 15.

B. subtilis amplicon NGS. Condition 4. Barcode sequence indicated by the orange region. Upper graph: percentage of reads showing a specific deletion/insertion. Lower graph: percentage of reads showing a specific base pair change. ~83200 read depth.

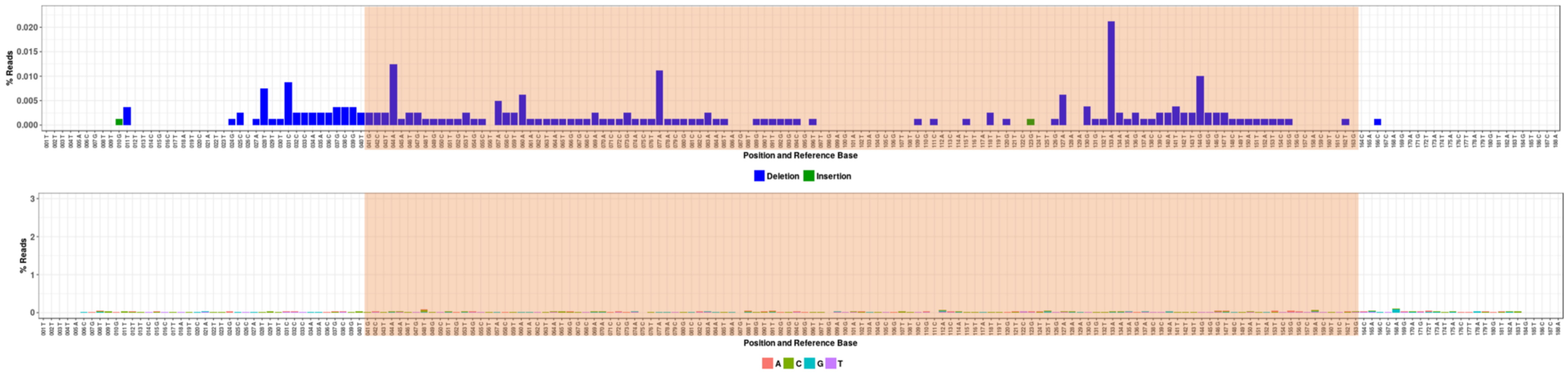

Supplementary Fig. 16.

P. putida amplicon NGS. Glycerol control. Barcode sequence indicated by the orange region. Upper graph: percentage of reads showing a specific deletion/insertion. Lower graph: percentage of reads showing a specific base pair change. ~79900 read depth.

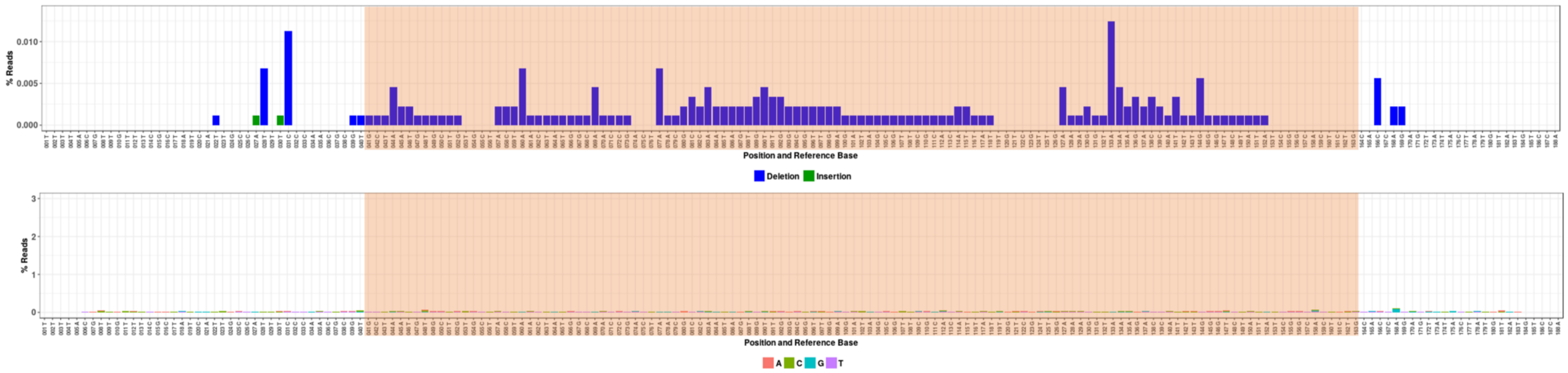

Supplementary Fig. 17.

P. putida amplicon NGS. Condition 1. Barcode sequence indicated by the orange region. Upper graph: percentage of reads showing a specific deletion/insertion. Lower graph: percentage of reads showing a specific base pair change. ~88300 read depth.

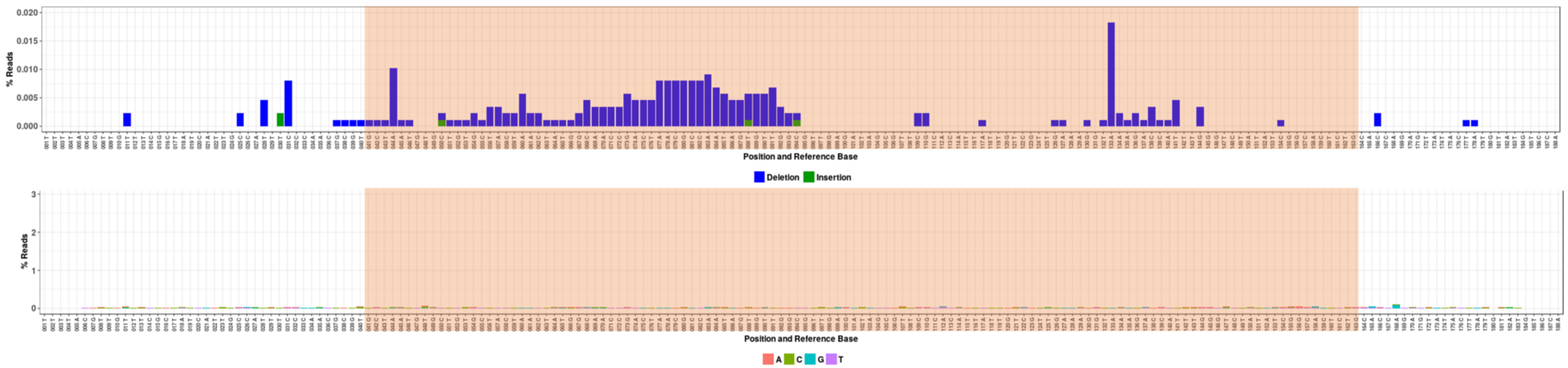

Supplementary Fig. 18.

P. putida amplicon NGS. Condition 2. Barcode sequence indicated by the orange region. Upper graph: percentage of reads showing a specific deletion/insertion. Lower graph: percentage of reads showing a specific base pair change. ~87400 read depth.

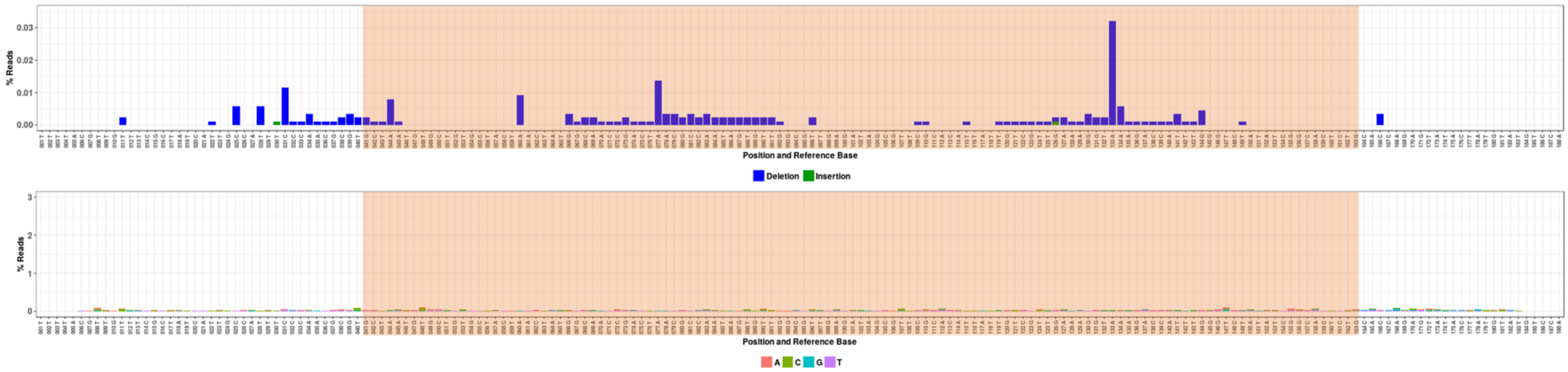

Supplementary Fig. 19.

P. putida amplicon NGS. Condition 3. Barcode sequence indicated by the orange region. Upper graph: percentage of reads showing a specific deletion/insertion. Lower graph: percentage of reads showing a specific base pair change. ~86800 read depth.

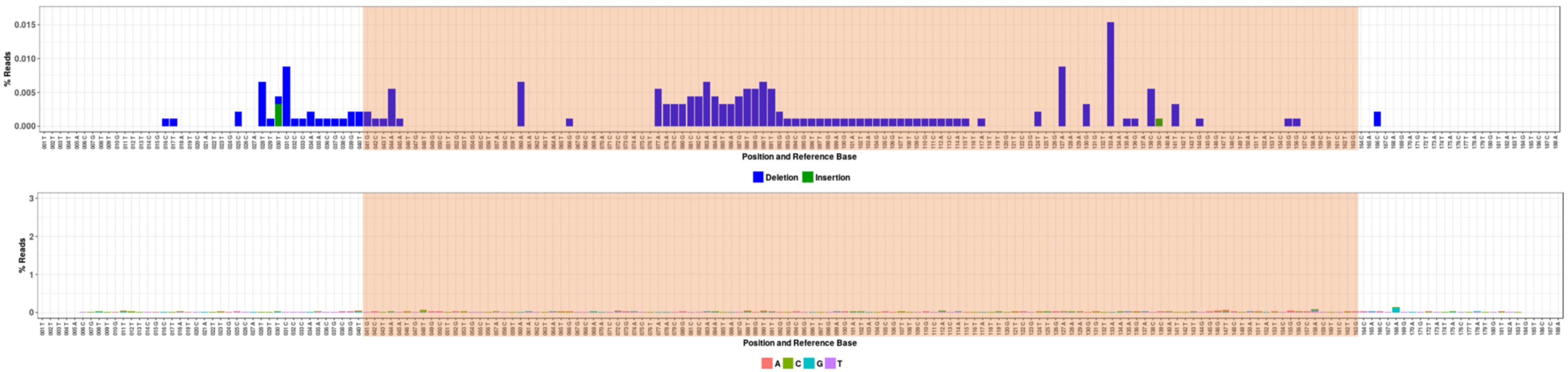

Supplementary Fig. 20.

P. putida amplicon NGS. Condition 4. Barcode sequence indicated by the orange region. Upper graph: percentage of reads showing a specific deletion/insertion. Lower graph: percentage of reads showing a specific base pair change. ~90800 read depth.

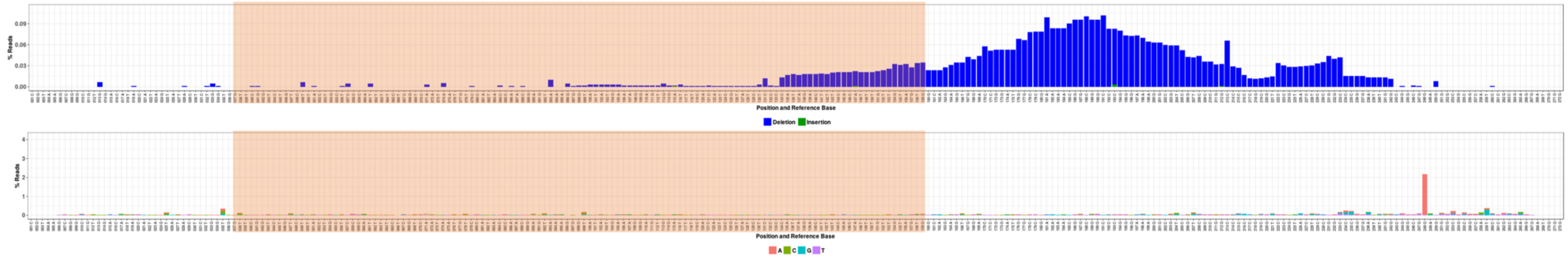

Supplementary Fig. 21.

S. albidoflavus amplicon NGS. Glycerol control. Barcode sequence indicated by the orange region. Upper graph: percentage of reads showing a specific deletion/insertion. Lower graph: percentage of reads showing a specific base pair change. ~87400 read depth.

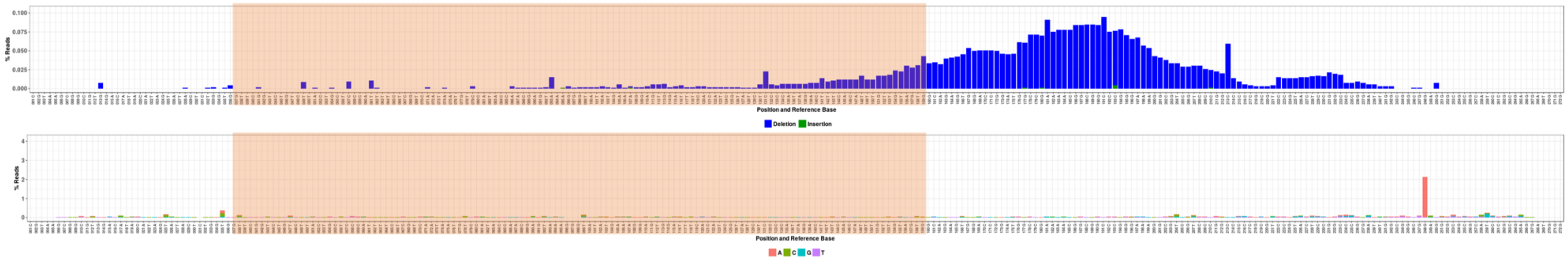

Supplementary Fig. 22.

S. albidoflavus amplicon NGS. Condition 1. Barcode sequence indicated by the orange region. Upper graph: percentage of reads showing a specific deletion/insertion. Lower graph: percentage of reads showing a specific base pair change. ~91700 read depth.

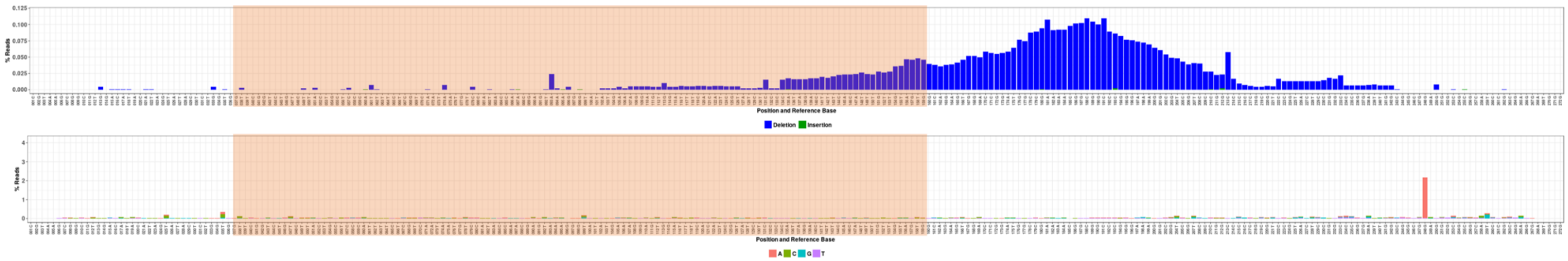

Supplementary Fig. 23.

S. albidoflavus amplicon NGS. Condition 2. Barcode sequence indicated by the orange region. Upper graph: percentage of reads showing a specific deletion/insertion. Lower graph: percentage of reads showing a specific base pair change. ~96800 read depth.

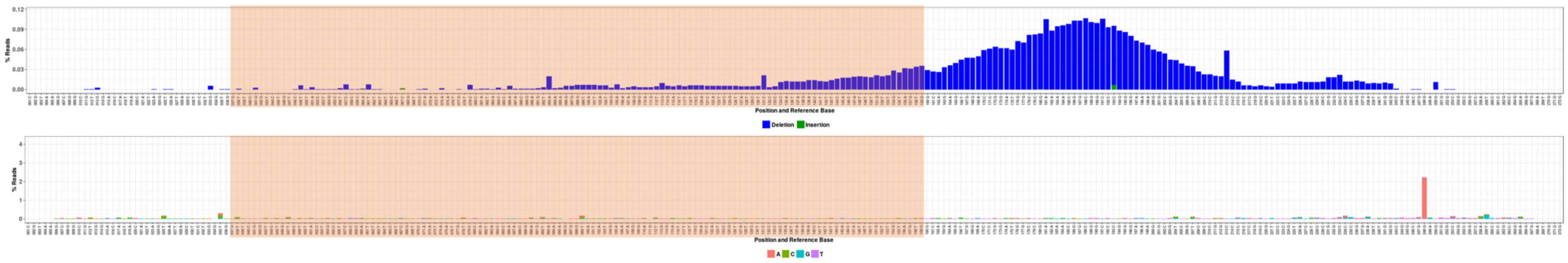

Supplementary Fig. 24.

S. albidoflavus amplicon NGS. Condition 3. Barcode sequence indicated by the orange region. Upper graph: percentage of reads showing a specific deletion/insertion. Lower graph: percentage of reads showing a specific base pair change. ~128500 read depth.

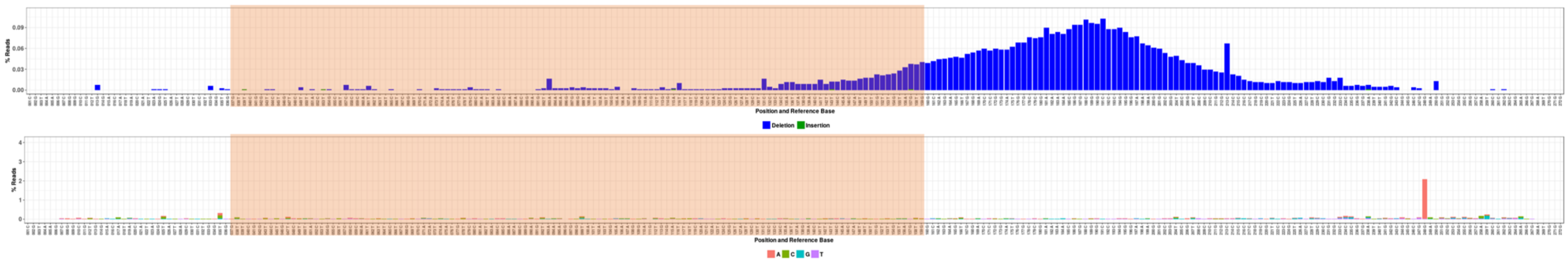

Supplementary Fig. 25.

S. albidoflavus amplicon NGS. Condition 4. Barcode sequence indicated by the orange region. Upper graph: percentage of reads showing a specific deletion/insertion. Lower graph: percentage of reads showing a specific base pair change. ~78100 read depth.

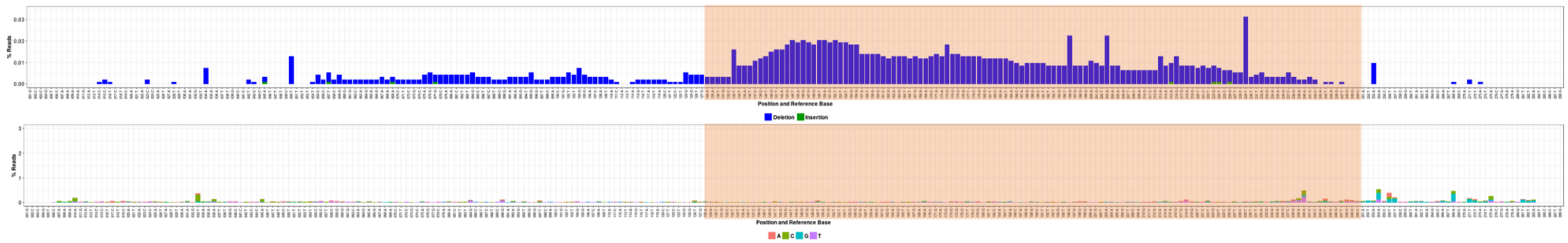

Supplementary Fig. 26.

S. cerevisiae amplicon NGS. Glycerol control. Barcode sequence indicated by the orange region. Upper graph: percentage of reads showing a specific deletion/insertion. Lower graph: percentage of reads showing a specific base pair change. ~91500 read depth.

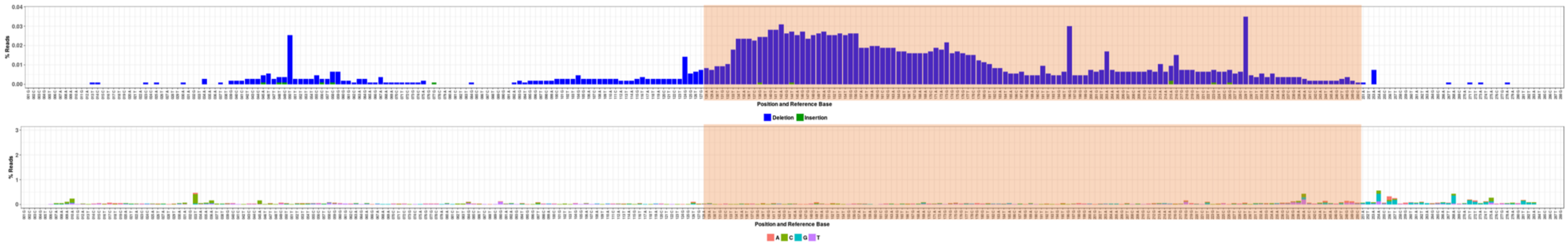

Supplementary Fig. 27.

S. cerevisiae amplicon NGS. Condition 1. Barcode sequence indicated by the orange region. Upper graph: percentage of reads showing a specific deletion/insertion. Lower graph: percentage of reads showing a specific base pair change. ~105000 read depth.

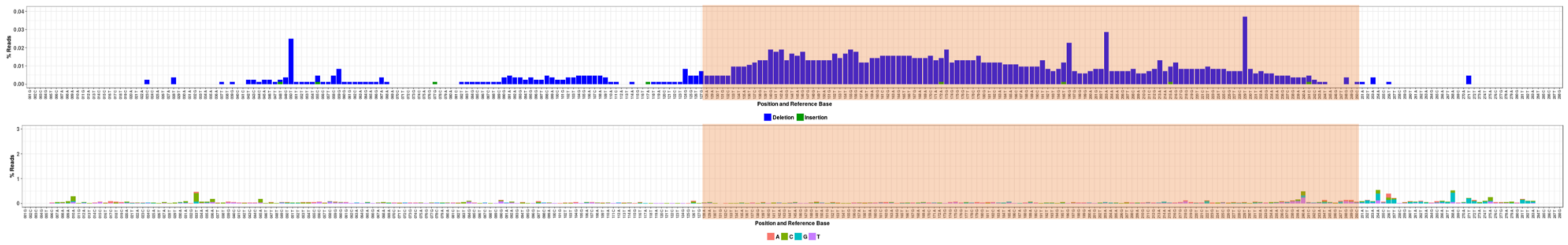

Supplementary Fig. 28.

S. cerevisiae amplicon NGS. Condition 2. Barcode sequence indicated by the orange region. Upper graph: percentage of reads showing a specific deletion/insertion. Lower graph: percentage of reads showing a specific base pair change. ~82600 read depth.

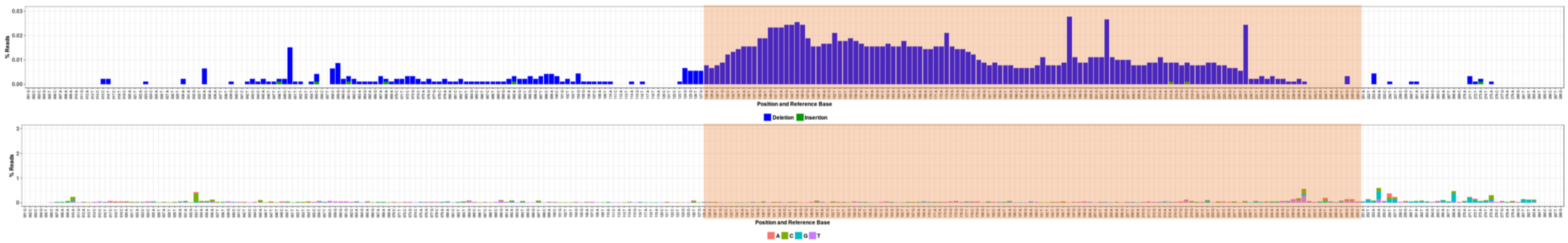

Supplementary Fig. 29.

S. cerevisiae amplicon NGS. Condition 3. Barcode sequence indicated by the orange region. Upper graph: percentage of reads showing a specific deletion/insertion. Lower graph: percentage of reads showing a specific base pair change. ~89000 read depth.

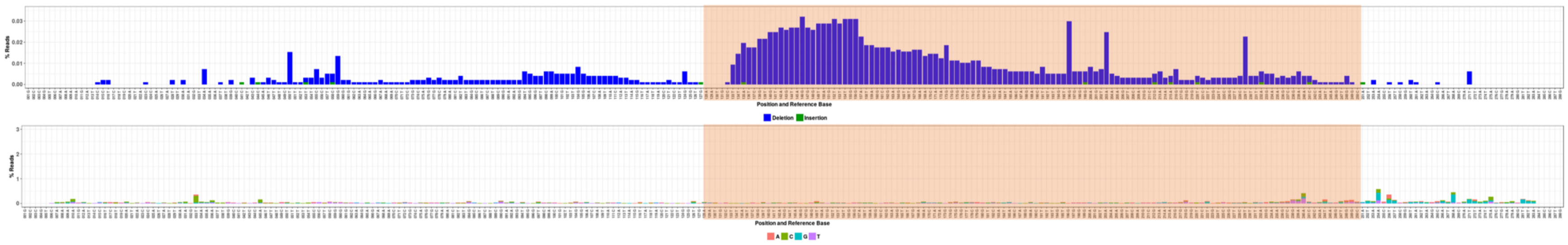

Supplementary Fig. 30.

S. cerevisiae amplicon NGS. Condition 4. Barcode sequence indicated by the orange region. Upper graph: percentage of reads showing a specific deletion/insertion. Lower graph: percentage of reads showing a specific base pair change. ~95500 read depth.

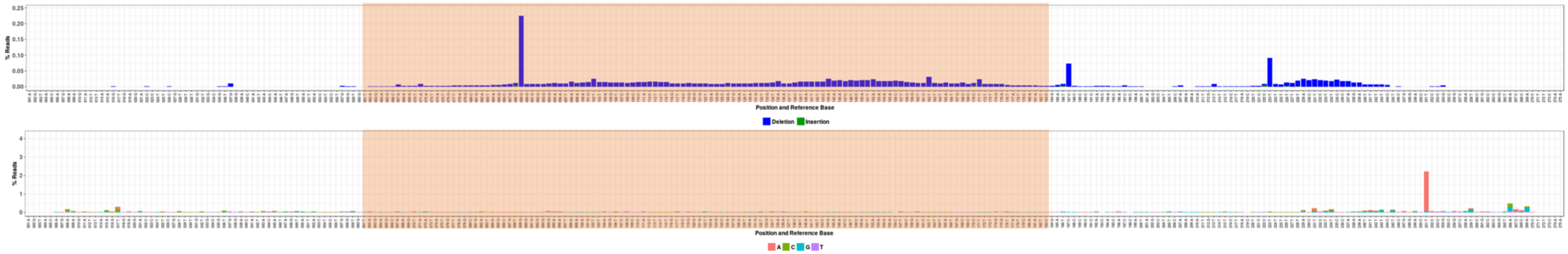

Supplementary Fig. 31.

K. phaffii amplicon NGS. Glycerol control. Barcode sequence indicated by the orange region. Upper graph: percentage of reads showing a specific deletion/insertion. Lower graph: percentage of reads showing a specific base pair change. ~73000 read depth.

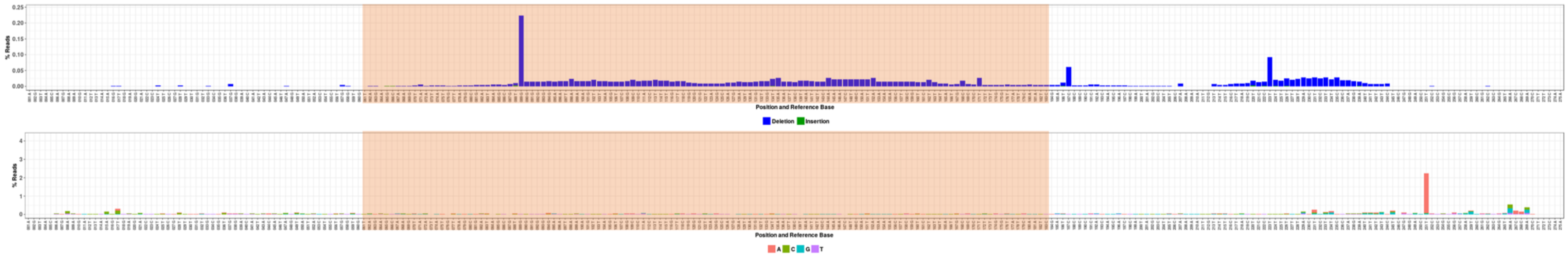

Supplementary Fig. 32.

K. phaffii amplicon NGS. Condition 1. Barcode sequence indicated by the orange region. Upper graph: percentage of reads showing a specific deletion/insertion. Lower graph: percentage of reads showing a specific base pair change. ~82900 read depth.

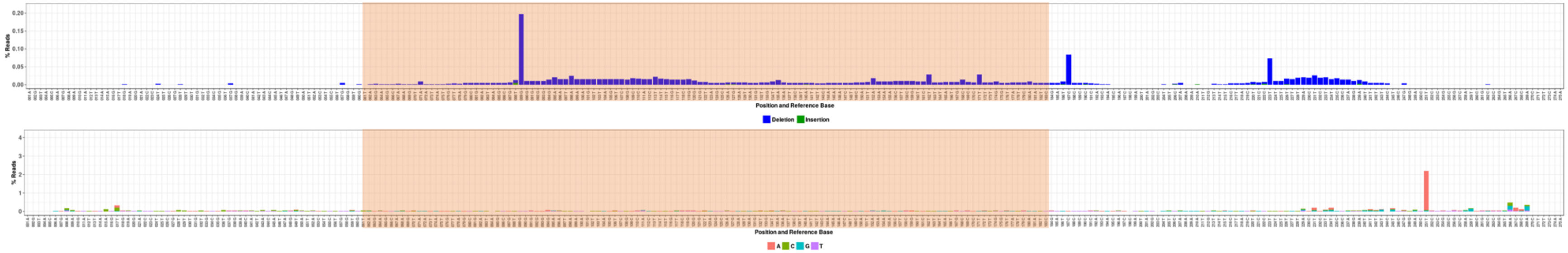

Supplementary Fig. 33.

K. phaffii amplicon NGS. Condition 2. Barcode sequence indicated by the orange region. Upper graph: percentage of reads showing a specific deletion/insertion. Lower graph: percentage of reads showing a specific base pair change. ~85400 read depth.

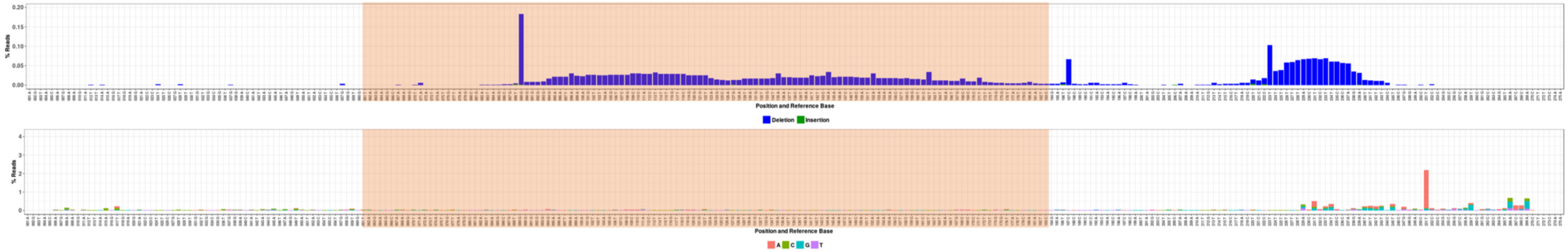

Supplementary Fig. 34.

K. phaffii amplicon NGS. Condition 3. Barcode sequence indicated by the orange region. Upper graph: percentage of reads showing a specific deletion/insertion. Lower graph: percentage of reads showing a specific base pair change. ~90600 read depth.

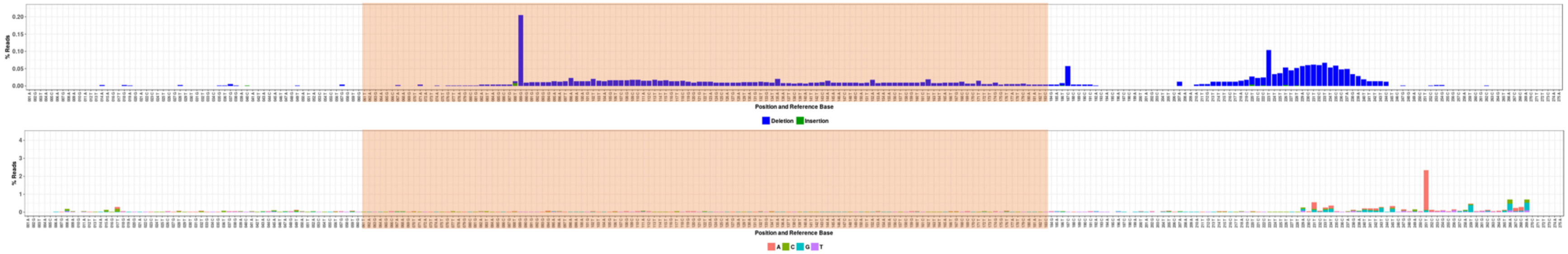

Supplementary Fig. 35.

K. phaffii amplicon NGS. Condition 4. Barcode sequence indicated by the orange region. Upper graph: percentage of reads showing a specific deletion/insertion. Lower graph: percentage of reads showing a specific base pair change. ~84600 read depth.

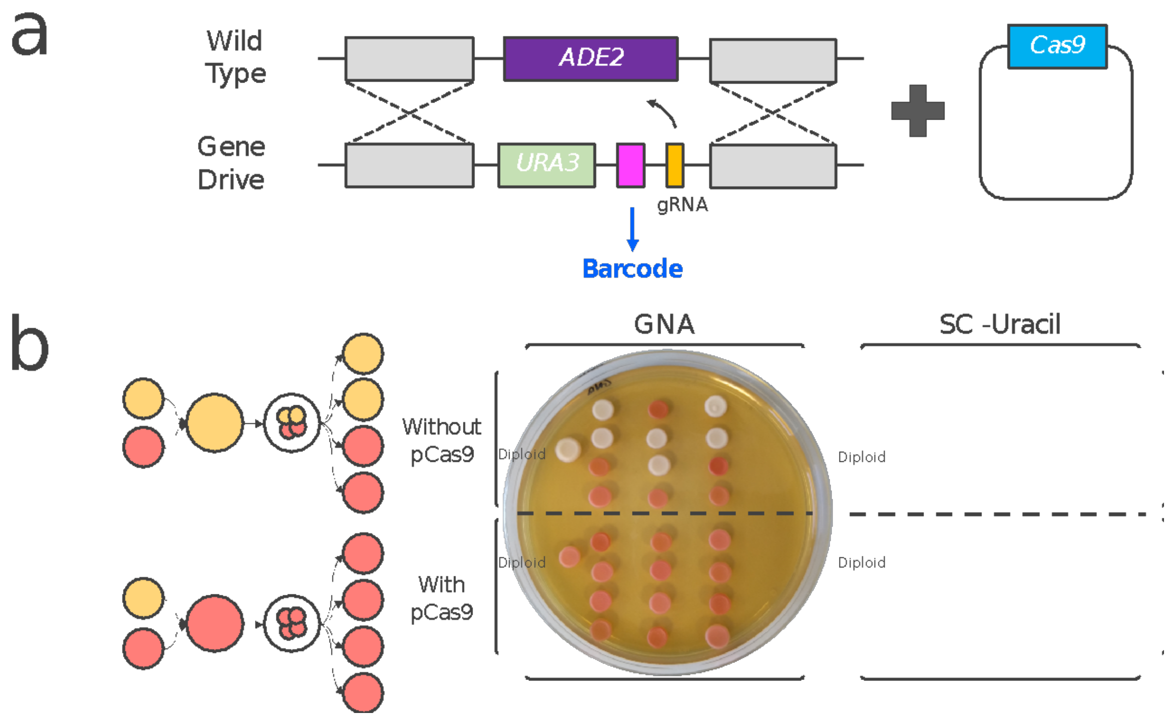

Supplementary Fig. 36.

Gene drive experiment in *S. cerevisiae*. a) Graphic representation of the gene drive mechanism used to propagate a barcode. A haploid strain (gene drive) containing a deletion of *ADE2* gene substituted by *URA3* marker, the barcode sequence and a gRNA targeting *ADE2* is mated with a wild type strain of the other sex. In the presence of a plasmid carrying the Cas9 gene, the CRISPR machinery edits the *ADE2* gene propagating the desired barcoded phenotype (red colonies, uracil auxotrophic). b) Yeast gene drive experiment results. Haploid cells containing the gene drive cassette were mated with wild type cells in the presence of Cas9. The diploid cells resulting from this were allowed to sporulate and the tetrads were dissected. When Cas9 was not present, the barcoded cassette was inherited by 50% of the final haploid population. When Cas9 was present, the drive allowed the copy of *URA3* gene and the barcode DNA in all the spores.

Supplementary Table 1.

Putative essential gene list of *S. albidoflavus*. Locus tag names from NCBI accession number CP004370.1.

XNR_0060	XNR_4623	XNR_2921	XNR_1124
XNR_5409	XNR_4512	XNR_2567	XNR_1072
XNR_5408	XNR_4358	XNR_2566	XNR_1004
XNR_5406	XNR_4333	XNR_3654	XNR_0818
XNR_5375	XNR_4331	XNR_3700	XNR_0712
XNR_5370	XNR_5405	XNR_3750	XNR_0710
XNR_5365	XNR_1843	XNR_3763	XNR_0462
XNR_5364	XNR_1908	XNR_3772	XNR_0461
XNR_5357	XNR_2086	XNR_3784	XNR_4832
XNR_5356	XNR_2129	XNR_3870	XNR_0281
XNR_5355	XNR_3512	XNR_3872	XNR_0772
XNR_5354	XNR_3475	XNR_4145	XNR_1514
XNR_5276	XNR_3473	XNR_1479	XNR_5582
XNR_5171	XNR_3434	XNR_1247	XNR_5368
XNR_5062	XNR_3431	XNR_1238	XNR_5367
XNR_5040	XNR_2759	XNR_1196	XNR_0715
XNR_4886	XNR_3194	XNR_1164	XNR_0714
XNR_4775	XNR_3034	XNR_1141	

Supplementary Table 2.

Plasmids used in this study. Restriction sites used to linearize the vectors are shown in italics. (*) Two different plasmids were built with two sgRNA sequences.

Target species	Task	Plasmid Name	Markers	Information	Reference
E. coli	λ-Red	pKD46	Ampicillin	λ-red genes under pBad promoter, temperature-sensitive	[1]
		pCP20	Ampicillin / Chloramphenicol	FLP recombinase, temperature-sensitive	[1]
		pKD4	Ampicillin / Kanamycin	R6K-γ ori, Kanamycin resistance gene between FRT sequences	[1]
		pEC-Red2	Ampicillin / Chloramphenicol	From pKD4. cat gene between FRT sites, homologous arms. BamHI	This study
		pEC-Red2-BC	Ampicillin / Chloramphenicol	Barcoded pEC-Red2	This study
	CRISPR	pREDCas9	Spectinomycin	Constitutive Cas9 expression, λ-red genes under IPTG inducible promoter, sgRNA targeting pUC origin under pBAD promoter, temperature-sensitive replicon	[2]
		pEC-CRISPR2	Ampicillin	pUC origin, gRNA and homologous arms targeting barcoding region. SphI	This study
		pEC-CRISPR2-BC*	Ampicillin	Barcoded pEC-CRISPR2	This study
B. subtilis	Cre-Lox	pBS-CreLox2	Spectinomycin / Zeocin	ZeoR gene between loxP sites and homologous arms. SpeI	This study
		pBS-CreLox2-BC	Spectinomycin / Zeocin	A barcoded version of pBS-CreLox2	This study
		pDR244	Spectinomycin / Ampicillin	Cre recombinase expression, temperature-sensitive.	[5]
	CRISPR	pJOE8999.1	Kanamycin	Cas9 under mannose inducible promoter, sgRNA under constitutive promoter, temperature-sensitive	[6]
		pBS-CRISPR2	Kanamycin	From pJOE8999.1. sgRNA under strong pVeg promoter, homologous arms, temperature-sensitive. SpeI	This study
		pBS-CRISPR2-BC	Kanamycin	From pBS-CRISPR2. Barcoded.	This study
P. putida	CRISPR/targetron	pSEVA421-Cas9tr	Streptomycin/ Spectinomycin	pSEVA421 derivative bearing the cas9 gene and tracrRNA; oriV (RK2)	[10]
		pSEVA231-C-94a	Kanamycin	pSEVA231 derivative bearing the CRISPR array and spacer	[9,10]

				to counterselect intron-barcode insertion; oriV (pBBR1)	
		pSEVA6511-GIIi	Gentamycin	Ll.LtrB expression plasmid under control of ChnR/pChnB promoter.; oriV(RSF1010)	Velázquez et al, in preparation.
		pSEVA6511-GIIi-BC	Gentamycin	pSEVA6511-GIIi derivative with Barcode inserted in MluI site inside Ll.LtrB	This study
S. albidoflavus	CRISPR	pCRISPomyces-2	Apramycin	Constitutive expression of Cas and sgRNA	[13]
		pSA-CRISPR	Apramycin	From pCRISPomyces-2. Homologous regions and gRNA for barcoding. SpeI	This study
		pSA-CRISPR-BC*	Apramycin	Barcoded pSA-CRISPR	This study
S. cerevisiae	Cre-Lox	pCfB2899	Ampicillin	Homologous regions targeting Chr X. ADH1 and CYC1 terminators between arms.	[16]
		pSC	Ampicillin	From pCfB2899. Terminators removed. EcoRI	This study
		pSC-CreLox	Ampicillin / KanMX	From pSC. KanMX (also *URA3* variant) between loxP sites. EcoRI	This study
		pSC-CreLox-BC	Ampicillin / KanMX	Barcoded pSC-CreLox	This study
		pBF3060	Ampicillin / *URA3*	CreA gene under GAL1 promoter	[15]
		pBF3060_NatMX	Ampicillin / NatMX	From pBF3060. *URA3* subsituted by NatMX	This study
	CRISPR	pCfBf2312	Ampicillin / KanMX	Cas9 under TEF1 promoter	[16]
		pCfBf3020	Ampicillin / NatMX	sgRNA under SNR52 promoter	[16]
		pSC-CRISPR-BC	Ampicillin	From pSC. Barcoded.	This study
	Gene Drive	pGD-ADE2	Ampicillin / *URA3*	*ADE2* homologous regions, sgRNA under SNR52 promoter, *URA3*, barcoded.	This study
		pXP622	Ampicillin / *LEU2*	*LEU2* marker	[15]
K. phaffii	*AOX1* insertion	pICXNH3	Zeocin / Chloramphenicol	ccdB and Cat genes between *AOX1* promoter and terminator	[17]
		pPP	Zeocin	From pICXNH3, ccdB and Cat removed. BamHI	This study
		pPP-BC	Zeocin	From pPP, barcoded	This study

Supplementary Table 3.

Barcoding site description and barcode sequence information.

Species (strain)	NCBI accession	Left gene (orientation)	Right gene (orientation)	Barcode sequence
E. coli (BW25113)	CP009273.1	*pyrH* (+)	*frr* (+)	GTCGTGTCTGTCAATATCGGGGATAATAAATGTAGATAATGATTTCGTCAAGTCCTTACCCCGACTATACAACAGCAATCCATAGTAATCTGTGCGAATGGTTTAGTAGGACATAACACAAAT
B. subtilis (168)	AL009126.1	*rpsD* (+)	*tyrS* (-)	CTTACTATGCCACATAGTCTAACTACTTTGCGTAGGTAATAGAGCAACGAGTAGGGTTATCGTCTTTCATACTCGGTTGGCGATTATACAAACTCCAATGGTTTGATTGGTGTTTCTCATACG
P. putida (EM383)	AE015451.2 (modified)	*Hypothetical PP5408 (-)*	*glmS (-)*	CAGAGTGCCGATATAGACCCAAATGGTCTTTACCTTTCAACGACAATAATGTGCGAACGCTATCTCAACGCCAACACTATTGGCGTTAGTCGGTTGCCATAGTTAGTAGCACAGCACATTAGC
S. albidoflavus (J1074)	CP004370.1	*XNR_3871* (-)	*XNR_3872* (+)	GTTCGGTCTGTCTTACTGCTCCCATTTTCTCGTCAATAATGTCCATACCAACGAGTAAAGGGTATATGAAAGGAGTGGATTTCACATCGTATCTCCCCATAGACTTTCACATTTGTTGTAGTG
S. cerevisiae (BY4742)	CP026288.1	*NCA3* (-)	*ASF1* (+)	AATGCTTATCGTGTAGTAGGGTAGTTTGGATAGCGAGTTGAACAAGGGGTCGGTCATACAGACTTTGTGGGGAGTAAAGTCAGCGAAAGGGGTCTGTCGTCTTTAGAAGCGAACCATAGTGGC
				ATTGGGGTGGTTCGTCAATGTGTAGAGCGTTGTGAGTAAGTTATTGAGTTCAACGGGGCAAATCTTTATATTGGGGATAACTAAAGTCAATGCGAACCATAGTTGAACTAACTTGGGGGAGAG
K. phaffii (GS115)	FN392322	*AOX1* (-)	*PAO* (+)	TAGAGCAAGTAATATGTAGCGATAGCGTGGGGGGAATAAAGTTAGTCGCCACTTTGTCGGGTAGCAGATAGTGTAATCTATTCAAACTCATAAAACTCGTCTTTATGCCCTTTGGTATCAATC

Supplementary Table 4.

Primers used to sequence the barcoded PCR amplicon. The Illumina adapter sequences appear in bold.

Species	Primer	Sequence
E. coli	BC_482	**ACACTCTTTCCCTACACGACGCTCTTCCGATCT**CCGTGTGGTAATGGGTGAAA
	BC_483	**GACTGGAGTTCAGACGTGTGCTCTTCCGATCT**CGGGTAATCGTCTGGATTATTAGG
B. subtilis	BC_498	**ACACTCTTTCCCTACACGACGCTCTTCCGATCT**GCAATCGTACACATGAGATTCAC
	BC_499	**GACTGGAGTTCAGACGTGTGCTCTTCCGATCT**AGATCCTTTGCCACTGAAGG
P. putida	BC_500	**ACACTCTTTCCCTACACGACGCTCTTCCGATCT**CGATGACAATGAAAGCATACAACAAGAG
	BC_501	**GACTGGAGTTCAGACGTGTGCTCTTCCGATCT**ATATTTATTACGTGGCGACGCGT
S. albidoflavus	BC_502	**ACACTCTTTCCCTACACGACGCTCTTCCGATCT**CAGGAGGTTGCGATGAAAGA
	BC_503	**GACTGGAGTTCAGACGTGTGCTCTTCCGATCT**GAATCTGGGCGCTCATACC
S. cerevisiae	BC_504	**ACACTCTTTCCCTACACGACGCTCTTCCGATCT**CGGAGATGGCGCATCTATTT
	BC_505	**GACTGGAGTTCAGACGTGTGCTCTTCCGATCT**CCTCTGTGAGGCCGATTATG
K. phaffii	BC_506	**ACACTCTTTCCCTACACGACGCTCTTCCGATCT**GGTATTTCCCACTCCTCTTCAG
	BC_507	**GACTGGAGTTCAGACGTGTGCTCTTCCGATCT**TATGCTGTGCTTGGGTGTT

Supplementary Table 5.

The efficiency of the described barcoding methods used in this article for each species. All the experiments were carried out in three different replicas. After transformation with the PCR product/plasmid colonies were boiled and used as PCR template. The table shows how many positive clones were obtained in all experiments out of the number of tested colonies.

**Species**	**Method**	**Replica 1**	**Replica 2**	**Replica 3**	**Average efficiency**
E. coli	λ-Red	7 / 8	7 / 8	8 / 8	92%
	CRISPR gRNA1	8 / 8	8 / 8	8 / 8	100%
	CRISPR gRNA2	8 / 8	7 / 8	7 / 8	92%
B. subtilis	Cre-Lox	8 / 8	7 / 8	8 / 8	96%
	T/AT	7 / 7	8 / 8	7 / 8	92%
	CRISPR	6 / 8	7 / 8	7 / 8	83%
P. putida	CRISPR/targetron	8/9	3/8	3/8	55%
S. albidoflavus	CRISPR gRNA1	5 / 6	2 / 4	5 / 5	77%
	CRISPR gRNA2	5 / 7	3 / 5	2 / 3	66%
S. cerevisiae	Cre-Lox	8 / 8	7 / 8	6 / 6	96%
	CRISPR	7 / 8	7 / 8	8 / 8	92%
K. phaffii	*AOX1* insertion	8 / 8	7 / 8	7 / 7	96%

Supplementary Table 6.

CellRepo links of the barcoded strains in this study.

Species	Link to CellRepo	QR code
E. coli	https://cellrepo.ico2s.org/repositories/59?branch_id=82&locale=en	
B. subtilis	https://cellrepo.ico2s.org/repositories/60?branch_id=85&locale=en	
P. putida	https://cellrepo.ico2s.org/repositories/61?branch_id=89&locale=en	
S. albidoflavus	https://cellrepo.ico2s.org/repositories/62?branch_id=91&locale=en	
S. cerevisiae	https://cellrepo.ico2s.org/repositories/65?branch_id=96&locale=en	
K. phaffii	https://cellrepo.ico2s.org/repositories/64?branch_id=94&locale=en	

Sequencing results:

P-values were automatically calculated by Geneious 2019 using binomial distribution as described in the user manual (https://assets.geneious.com/documentation/geneious/GeneiousPrimeManual2019.2.pdf).

Supplementary Table 7. Sequencing statistics (upper table) and mutation analysis (lower table) of *E. coli* clones barcoded using Lambda-Red and CRISPR (using two different gRNAs) and compared against BW25113 reference genome (CP009273.1).

Method	Strain	Length (bp)	Sequences	Identical sites	Pairwise identity	Coverage Mean	Coverage Std Dev	Covergage Min	Coverage Max
Lambda-Red	**1**	4,632,808	11,812,588	3,983,330 (86.0%)	99.90%	371.3	64.6	0	769
	**2**	4,633,255	14,409,073	3,841,995 (82.9%)	99.90%	451.6	72.8	0	953
	**3**	4,632,898	11,804,172	3,957,826 (85.4%)	99.90%	369.8	59.6	0	829
CRISPR sgRNA 1	**1**	4,632,569	9,274,681	4,125,954 (89.1%)	99.90%	291.2	46.6	0	707
	**2**	4,632,629	10,777,258	4,003,022 (86.4%)	99.90%	337.4	52.7	0	784
	**3**	4,632,851	11,579,032	4,046,097 (87.3%)	99.90%	363.9	56.2	0	809
CRISPR sgRNA 2	**1**	4,632,850	13,309,565	3,885,625 (83.9%)	99.90%	416.9	61.1	0	955
	**2**	4,632,556	9,193,478	4,079,340 (88.1%)	99.90%	288	45.5	0	653
	**3**	4,632,559	8,974,523	4,144,709 (89.5%)	99.90%	282.4	44.9	0	649

Method	Strain	Minimum	Maximum	Change	Type	Coverage	Variant frequency	P-value	CDS name	Codon Change	AA change	Protein Effect
Lambda-Red	**1**	1,952,708	1,952,708	C -> T	SNP (transition)	315	100.00%	0	Intergenic	---	---	---
	**2**	1,952,708	1,952,708	C -> T	SNP (transition)	341	100.00%	0	Intergenic	---	---	---
	**3**	1,952,708	1,952,708	C -> T	SNP (transition)	317	100.00%	0	Intergenic	---	---	---
CRISPR sgRNA 1	**1**	None										
	**2**	None										
	**3**	2,016,625	2,016,625	T -> G	SNP (transition)	297	100.00%	0	fliR CDS	CTT -> CTG		None
		3,531,572	3,531,572	G -> T	SNP (transition)	460	100.00%	0	yhgF CDS	GGC -> TGC	G -> C	Substitution
CRISPR sgRNA 2	**1**	None										
	**2**	None										
	**3**	None										

Supplementary Table 8.
Sequencing statistics (upper table) and mutation analysis (lower table) of *B. subtilis* clones barcoded using three different methods and compared against 168 reference genome (AL009126.1).

Method	Strain	Length	Sequences	Identical sites	Pairwise identity	Coverage Mean	Coverage Std Dev	Covergage Min	Coverage Max
Cre-Lox	**1**	4,217,349	10,795,770	3,601,061 (85.4%)	99.90%	369.3	53.2	0	1242
	**2**	4,217,213	9,229,938	3,724,674 (88.3%)	99.90%	316.8	54.6	0	1197
	**3**	4,217,457	10,391,000	3,714,984 (88.1%)	99.90%	357.5	58.3	0	994
CRISPR	**1**	4,217,215	10,496,279	3,366,442 (79.8%)	99.90%	357.7	41.8	0	1469
	**2**	4,217,330	11,354,246	3,426,110 (81.2%)	99.90%	388.9	43.7	0	1432
	**3**	4,217,375	10,318,738	3,443,869 (81.7%)	99.90%	352.8	41.2	0	1498
T/TA	**1**	4,217,314	11,466,520	3,381,751 (80.2%)	99.90%	392.2	43	0	1476
	**2**	4,217,454	13,153,649	3,275,819 (77.7%)	99.90%	449.3	52.5	0	1826
	**3**	4,217,545	11,992,540	3,365,142 (79.8%)	99.90%	410.2	47.2	0	1290

Method	Strain	Minimum	Maximum	Change	Type	Coverage	Variant frequency	P-value	CDS name	Codon Change	AA change	Protein Effect
Cre-Lox	**1**	2,123,739	2,123,739	T -> A	SNP (transversion)	289	77.50%	0	yojE CDS	ATA -> TTA	I -> L	Substitution
	**2**	None										
	**3**	2,625,604	2,625,604	G -> T	SNP (transversion)	312	100.00%	0	dnaJ CDS	GGC -> GGA		None
		2,932,230	2,932,230	G -> T	SNP (transversion)	378	100.00%	0	ysfB CDS	GTG -> GTT		None
CRISPR	**1**	None										
	**2**	None										
	**3**	None										
T/TA	**1**	None										
	**2**	None										
	**3**	None										

Supplementary Table 9.
Sequencing statistics (upper table) and mutation analysis (lower table) of *P. putida* clones barcoded CRISPR/targetron method and compared against EM383 reference genome (a modified version of AE015451.2).

**Method**	**Strain**	**Length**	**Sequences**	**Identical sites**	**Pairwise identity**	**Coverage Mean**	**Coverage Std Dev**	**Coverg age Min**	**Covera ge Max**
CRISPR/targetr on	**1**	5,913,473	13,587,360	4,824,315 (81.6%)	99.80%	332.8	39.8	4	1290
	**2**	5,913,390	12,875,395	4,925,599 (83.3%)	99.80%	316.1	34.2	8	1093

**Method**	**Strain**	**Minimum**	**Maximum**	**Change**	**Type**	**Coverage**	**Variant frequency**	**P-value**	**CDS name**	**Cod on Cha nge**	**AA chang e**	**Protei n Effect**
CRISPR/targetr on	**1**	3,047,795	3,047,881	- AGTCGCCCGGACTCTGGAGGCACCCCAAGGA TCGCGCCTGGCAATACAAGGATCTGGAATAC TGGACGGACCTGGCCAGGTTGCTTG	Deletion	253	100.00%	0.00E+0 0	dmoA-I	Deletion		
	**2**	1,078,836	1,078,835	(C)4 -> (C)5	Insertion (tandem repeat)	344	93.00%	0.00E+0 0	cafA CDS	Frame Shift		

Supplementary Table 10.
Sequencing statistics (upper table) and mutation analysis (lower table) of *S. cerevisiae* clones barcoded using two methods and compared against BY4742 reference genome (GenBank assembly accession: GCA_003086655.1).

Method	Strain	Length	Sequences	Identical sites	Pairwise identity	Coverage Mean	Coverage Std Dev	Covergage Min	Coverage Max
Cre-Lox	**1**	12,177,589	7,566,425	11,725,736 (96.3%)	99.90%	89.5	113.3	0	4841
	**2**	12,178,378	8,353,507	11,685,184 (96.0%)	99.90%	98.7	124.2	0	4972
	**3**	12,179,100	9,626,768	11,556,364 (94.9%)	99.90%	113.2	125.3	0	5208
CRISPR	**1**	12,177,604	6,969,487	11,721,633 (96.3%)	99.90%	82	117.6	0	3623
	**2**	12,180,145	9,615,749	11,597,471 (95.2%)	99.90%	113.4	161	0	6794
	**3**	12,178,498	7,698,946	11,697,279 (96.1%)	99.90%	90.6	131.7	0	4013

Method	Strain	Chromosome	Minimum	Maximum	Change	Type	Coverage	Variant frequency	P-value	CDS name	Codon Change	AA change	Protein Effect
Cre-Lox	**1**	None											
	**2**	X	343,105	343,105	A -> G	SNP (transition)	109	100.00%	0.00E+00	MTR4	GCA -> GCG		None
	**3**	X	11,273	11,272	(TA)13 -> (TA)15	Insertion (tandem repeat)	94 -> 95	61.7% -> 62.1%	6.80E-29	Intergenic	---	---	---
CRISPR	**1**	None											
	**2**	IX	11,243	11,246	(TA)14 -> (TA)12	Deletion (tandem repeat)	49	98.00%	4.70E-09	Intergenic	---	---	---
		IX	11,273	11,272	(TA)13 -> (TA)15	Insertion (tandem repeat)	183 -> 185	95.1% -> 95.7%	1.80E-41	Intergenic	---	---	---
		XV	1,208	1,209	(AT)12 -> (AT)11	Deletion (tandem repeat)	121 -> 123	83.7% -> 85.1%	8.40E-133	Intergenic	---	---	---
		XV	1,233	1,232	(AT)12 -> (AT)13	Insertion (tandem repeat)	132 -> 134	89.4% -> 89.6%	4.20E-289	Intergenic	---	---	---
	**3**	XVI	532,368	532,368	C -> G	SNP (transversi on)	81	100.00%	3.20E-284	Intergenic	---	---	---

Supplementary Table 11.
Sequencing statistics (upper table) and mutation analysis (lower table) of *K. phaffii* clones barcoded using *AOX1* insertion method and compared against GS115 reference genome (GenBank assembly accession: GCA_000027005.1).

Method	Strain	Length	Sequences	Identical sites	Pairwise identity	Coverage Mean	Coverage Std Dev	Coverage Min	Coverage Max
AOX1 insertion	**1**	9,227,849	13,649,028	8,237,869 (89.3%)	98.60%	214	76.5	0	7076
	**2**	9,222,446	8,752,994	8,714,000 (94.5%)	98.60%	137.1	45	0	3964

Method	Strain	Chromosome	Minimum	Maximum	Change	Type	Coverage	Variant frequency	P-value	CDS name	Codon Change	AA change	Protein Effect
AOX1 insertion	**1**	None											
	**2**	II	908,946	908,946	T -> A	SNP (transversion)	140	99.30%	0.00E+00	Intergenic	---	---	---
		III	81,689	81,689	T -> G	SNP (transversion)	152	100.00%	0.00E+00	Intergenic	---	---	---

Supplementary Table 12.
Sequencing statistics (upper table) and mutation analysis (lower table) of *S. albidoflavus* clones barcoded using gRNAs and compared against J1074 reference genome (CP004370.1).

**Method**	**Strain**	**Length**	**Sequences**	**Identical sites**	**Pairwise identity**	**Coverage Mean**	**Coverage Std Dev**	**Covergage Min**	**Coverage Max**
CRISPR sgRNA 1	**1**	6,845,309	11,542,819	5,975,030 (87.3%)	99.90%	241.7	61.5	0	6179
	**2**	6,844,965	9,789,335	6,151,432 (89.9%)	99.90%	206.1	47.1	0	5391
CRISPR sgRNA 2	**1**	6,844,139	6,756,753	6,336,488 (92.6%)	99.90%	142.1	33.7	0	3966
	**2**	6,844,905	11,429,476	6,011,854 (87.8%)	99.90%	239.5	49.1	0	5546
	**3**	6,846,031	13,416,563	5,964,405 (87.2%)	99.90%	281.9	56.2	0	5749

**Method**	**Strain**	**Minimum**	**Maximum**	**Change**	**Type**	**Coverage**	**Variant frequency**	**P-value**	**CDS name**	**Codon Change**	**AA change**	**Protein Effect**
CRISPR sgRNA 1	**1**	4,369,426	4,369,426	A -> G	SNP (transition)	250	100.00%	0.00E+00	XNR_3843	TTC -> CTC	F -> L	Substitution
		6,112,283	6,112,283	T -> C	SNP (transition)	220	99.50%	0.00E+00	XNR_5404	AAA -> GAA	K -> E	Substitution
	**2**	None										
CRISPR sgRNA 2	**1**	2,787,417	2,787,417	T -> G	SNP (transversion)	171	100.00%	0.00E+00	Intergenic	---	---	---
		3,586,859	3,586,859	T -> C	SNP (transition)	170	100.00%	0.00E+00	XNR_3149	GAA -> GGA	E -> G	Substitution
		4,127,513	4,127,513	A -> G	SNP (transition)	150	100.00%	0.00E+00	Intergenic	---	---	---
		4,712,156	4,712,156	C -> A	SNP (transversion)	133	100.00%	0.00E+00	XNR_4154	GCC -> TCC	A -> S	Substitution
		4,884,788	4,884,788	T -> C	SNP (transition)	133	100.00%	0.00E+00	Intergenic	---	---	---
		5,804,325	5,804,325	A -> G	SNP (transition)	115	100.00%	0.00E+00	Intergenic	---	---	---
	**2**	2,947,536	2,947,536	T -> C	SNP (transition)	258	100.00%	0.00E+00	XNR_2571	ATA -> GTA	I -> V	Substitution
		4,761,728	4,761,728	T -> C	SNP (transition)	159	80.50%	0.00E+00	Intergenic	---	---	---
		4,890,233	4,890,233	T -> C	SNP (transition)	304	99.70%	0.00E+00	XNR_4299	TTT -> TTC	---	None
	**3**	1,502,512	1,502,512	A -> G	SNP (transition)	347	61.10%	0.00E+00	XNR_1249	TTT -> TTC	---	None
		2,259,838	2,259,837	(G)10 -> (G)11	Insertion (tandem repeat)	188	53.70%	8.10E-269	Intergenic	---	---	---

Supplementary Table 13.

Sanger sequencing results of colonies after the 10 days or passes of each long-term survivability condition. Number of sequences identical to the barcode sequence/number of high-quality Sanger reactions retrieved. Important note: this was a preliminary experiment before NGS amplicon analysis, no replicas were sent for each sequencing reaction. This may explain low-quality reads and point mutations found. The discrepancies were single point mutations in all cases.

**Species**	**Condition 1**	**Condition 2**	**Condition 3**	**Condition 4**	**Condition 5**
E. coli	4/5	5/5	5/5	4/4	3/3
B. subtilis	5/5	5/5	3/3	4/4	3/3
P. putida	4/5	3/4	4/5	4/5	3/3
S. albidoflavus	2/2	2/3	4/4	4/4	3/3
S. cerevisiae	5/5	5/5	3/4	5/5	3/3
P. pastoris	5/5	5/5	5/5	4/4	2/3

References

1. Datsenko, K. A., Wanner, B. L. & Beckwith, J. One-step inactivation of chromosomal genes in Escherichia coli K-12 using PCR products. *Proc. Natl. Acad. Sci. U. S. A.* **97**, 6640–6645 (2000).
2. Li, Y. *et al.* Metabolic engineering of Escherichia coli using CRISPR–Cas9 meditated genome editing. *Metab. Eng.* **31**, 13–21 (2015).
3. Labun, K. *et al.* CHOPCHOP v3: expanding the CRISPR web toolbox beyond genome editing. *Nucleic Acids Res.* **47**, W171–W174 (2019).
4. Lin, Z. *et al.* A versatile mini-mazF-cassette for marker-free targeted genetic modification in Bacillus subtilis. *J. Microbiol. Methods* **95**, 207–214 (2013).
5. Koo, B. M. *et al.* Construction and Analysis of Two Genome-Scale Deletion Libraries for Bacillus subtilis. *Cell Syst.* **22**, 291–305 (2017).
6. Altenbuchner, J. Editing of the Bacillus subtilis Genome by the CRISPR-Cas9 System. *Appl. Environ. Microbiol.* **82**, 5421–7 (2016).
7. Priceid, M. A., Cruz, R., Baxter, S., Escalettes, F. & Rosser, S. J. CRISPR-Cas9 In Situ engineering of subtilisin E in Bacillus subtilis. *PLoS One* **14**, e0210121 (2019).
8. Velázquez, E., Lorenzo, V. de & Al-Ramahi, Y. Recombination-Independent Genome Editing through CRISPR/Cas9-Enhanced TargeTron Delivery. *ACS Synth. Biol.* **8**, 2186–2193 (2019).
9. Velázquez, E., Al-Ramahi, Y., Tellechea, J., Krasnogor, N. & de Lorenzo, V. Targetron-Assisted Delivery of Exogenous DNA Sequences into Pseudomonas putida through CRISPR-Aided Counterselection. A*CS Synth. Biol.* **10**, 2552-2565 (2021)
10. Aparicio, T., de Lorenzo, V. & Martínez-García, E. CRISPR/Cas9-Based Counterselection Boosts Recombineering Efficiency in *Pseudomonas putida*. *Biotechnol. J.* **13**, 1700161 (2018).
11. Choi, K.-H. & Schweizer, H. P. mini-Tn7 insertion in bacteria with single attTn7 sites: example Pseudomonas aeruginosa. *Nat. Protoc.* **1**, 153–161 (2006).
12. Kessler, B., de Lorenzo, V. & Timmis, K. N. A general system to integratelacZ fusions into the chromosomes of gram-negative eubacteria: regulation of thePm promoter of theTOL plasmid studied with all controlling elements in monocopy. *Mol. Gen. Genet. MGG* **233**, 293–301 (1992).
13. Cobb, R. E., Wang, Y. & Zhao, H. High-Efficiency Multiplex Genome Editing of Streptomyces Species Using an Engineered CRISPR/Cas System. **18**, 3 (2018).
14. Kieser, T., Bibb, M. J., Buttner, M. J., Chater, K. F. & Hopwood, D. A. *Practical Streptomyces Genetics*.

(The John Innes Foundation, 2000).
15. Fang, F. *et al.* A vector set for systematic metabolic engineering in Saccharomyces cerevisiae. *Yeast* **28**, 123–136 (2011).
16. Jessop-Fabre, M. M. *et al.* EasyClone-MarkerFree: A vector toolkit for marker-less integration of genes into Saccharomyces cerevisiae via CRISPR-Cas9. *Biotechnol. J* **11**, 1110–1117 (2016).
17. Geertsma, E. R. & Dutzler, R. A Versatile and Efficient High-Throughput Cloning Tool for Structural Biology. *Biochemistry* **50**, 3272–3278 (2011).
